# Liposomal Delivery of Hepatoprotective Phytochemicals for Liver Diseases: Advances in Formulation, Targeted Delivery, and Clinical Translation

**DOI:** 10.3390/biomedicines14091946

**Published:** 2026-08-29

**Authors:** Dignesh Khunt, Jigna Khasiya, Sanjay Chauhan, Bhupendra G. Prajapati, Udaykumar Vegad, Sagar Salave

**Affiliations:** 1 School of Pharmacy, Gujarat Technological University, Gandhinagar 382028, India; 2 Department of Pharmaceutics, Parul Institute of Pharmacy, Faculty of Pharmacy, Parul University, Waghodia, Vadodara 391760, India; bhupen27@gmail.com; 3 Centre for Research Impact & Outcome, Chitkara College of Pharmacy, Chitkara University, Rajpura 140401, India; 4 National Institute of Pharmaceutical Education and Research (NIPER), Ahmedabad 382355, India

**Keywords:** liposomes, hepatoprotective phytochemicals, liver-targeted drug delivery, hepatic stellate cell targeting, non-alcoholic fatty liver disease

## Abstract

**Background/Objectives:** Chronic liver diseases (CLDs), including viral hepatitis, alcohol-related liver disease, non-alcoholic fatty liver disease, hepatic fibrosis, and hepatocellular carcinoma, account for approximately two million deaths annually and remain a global health challenge. Although phytochemicals exhibit antioxidant, anti-inflammatory, antifibrotic, and metabolic regulatory activities through modulation of Nrf2/Keap1, NF-κB/MAPK, TGF-β/Smad, and lipid metabolism pathways, their therapeutic translation is hindered by poor aqueous solubility, extensive first-pass metabolism, and low oral bioavailability. This review evaluates the potential of liposomal delivery systems to overcome these limitations and improve hepatic drug targeting. **Methods:** A structured narrative review was conducted using systematic literature search principles. PubMed/MEDLINE, Scopus, and Web of Science databases were searched for studies published between January 2000 and March 2025. Original research articles evaluating liposomal formulations of hepatoprotective phytochemicals were assessed with emphasis on formulation strategies, pharmacokinetics, therapeutic efficacy, targeting approaches, and translational potential. **Results:** Liposomal encapsulation frequently improved systemic exposure and, in several preclinical studies, increased oral bioavailability relative to free phytochemicals, although the magnitude of improvement varied substantially according to the compound, formulation, route of administration, and experimental model. Liposomal encapsulation also improved formulation stability and enabled controlled release in several studies. Surface engineering using polyethylene glycol and receptor-specific ligands, including galactose, lactobionic acid, glycyrrhetinic acid, and vitamin A, further improved circulation time and cell-specific hepatic delivery. Emerging technologies such as microfluidic manufacturing, biomimetic liposomes, and multifunctional formulations show promise for improving formulation reproducibility and therapeutic performance, although clinical evidence remains limited. **Conclusions:** Liposomal delivery represents a promising strategy for enhancing the pharmacokinetic performance and therapeutic efficacy of hepatoprotective phytochemicals. However, additional well-designed clinical studies, scalable manufacturing approaches, and regulatory standardization are required to facilitate successful clinical translation.

## 1. Introduction

Liver diseases remain a significant global health problem, accounting for an estimated two million deaths annually and encompassing a broad range of conditions including viral hepatitis (HBV and HCV), alcohol-related liver disease, non-alcoholic fatty liver disease and its more severe form, non-alcoholic steatohepatitis, drug-induced liver injury, and hepatocellular carcinoma [1,2]. Because the liver is central to detoxification, intermediary metabolism, bile production, and immune regulation, it is particularly susceptible to injury, and even minor subclinical damage can profoundly disturb physiological homeostasis [2]. Chronic liver damage has been demonstrated to trigger a vicious cycle of inflammatory activation, oxidative stress and fibrogenic signalling, which can over time lead to cirrhosis or malignant transformation; notably, the resulting immune and inflammatory microenvironment appears to converge across diverse non-cancer aetiologies once end-stage disease is reached, with cytokines such as IL-15 emerging as prognostic markers of outcome [3]. Although significant progress has been made in understanding the molecular mechanisms of hepatic injury, there are still few drugs available for the treatment of most chronic liver diseases, and the side-effect profiles of current drugs often limit their long-term use, highlighting the ongoing need for hepatoprotective drugs with significant efficacy and a favorable safety profile [4].

Botanical remedies have been a significant source of hepatoprotective agents in various traditional medicine systems throughout history, such as Ayurveda, Traditional Chinese Medicine, and Unani, which continues to guide modern pharmacological research. A variety of plant-derived secondary metabolites have been found to possess multi-target hepatoprotective activity, which is mediated by various mechanisms such as activation of the antioxidant response element (ARE) of the transcription factor NF-E2-related factor 2 (Nrf2), inhibition of NF-κB-mediated pro-inflammatory cytokine production, inhibition of fibrogenic TGF-β1/Smad2/3 signalling, and modulation of hepatocellular lipid metabolism via PPAR-α and SREBP-1 [5,6]. Silymarin from Silybum marianum, curcumin from *Curcuma longa*, resveratrol from *Polygonum cuspidatum*, quercetin, naringenin, berberine and glycyrrhizin are among the most extensively studied plant-derived agents and have demonstrated appreciable hepatoprotective effects in preclinical models and, in some cases, in early clinical studies. It should be noted that these agents are members of different phytochemical families, such as silymarin (flavonolignan), quercetin (flavonoid), curcumin (diarylheptanoid), resveratrol (stilbenoid) and berberine (protoberberine alkaloid), which is relevant to their formulation behaviour and hepatic pharmacokinetics [7].

Although the pharmacological properties of these phytochemicals are promising, their clinical application is significantly hindered by several biopharmaceutical limitations, which are well documented in the literature. The majority of polyphenolic and terpenoid hepatoprotective agents are poorly soluble in gastrointestinal fluids and are susceptible to chemical and enzymatic degradation in the acidic gastric environment, further limiting the fraction of absorbed drug that reaches the systemic circulation in pharmacologically active form. The oral bioavailability of curcumin and resveratrol has been reported to be consistently <1% while that of silymarin has been reported to range from 20 to 50% depending on the formulation used. The limitations are compounded by rapid plasma clearance, which limits the time of hepatic drug exposure after even modest oral absorption. These pharmacokinetic barriers combine to make it difficult to achieve clinically relevant drug levels at the site of hepatic damage, and render many otherwise powerful botanical drugs clinically ineffective in their natural form [8].

To overcome these formulation-related challenges, various advanced drug delivery platforms have been investigated in recent years, such as solid lipid nanoparticles, polymeric nanoparticles, nanoemulsions, self-emulsifying drug delivery systems, niosomes, and dendrimers, which have different advantages in terms of solubility enhancement, metabolic protection, or controlled release behaviour. Among these platforms, liposomes have the most extensive regulatory precedent, with multiple approved products across oncology and anti-infective indications. It should be stated at the outset that this regulatory maturity belongs to the platform and not to liposomal phytochemical products for liver disease, for which no formulation has advanced beyond Phase I. As phospholipid bilayer vesicles typically ranging from 50 to 400 nm in diameter, liposomes are capable of simultaneously encapsulating hydrophilic compounds within their aqueous core and lipophilic compounds within the bilayer membrane, a structural versatility that is especially valuable for plant-derived agents whose physicochemical properties span a wide range of aqueous solubility and partition coefficient values. Furthermore, the phospholipid composition of liposomes confers a structural resemblance to biological cell membranes that facilitates cellular uptake while limiting the carrier-associated cytotoxicity concerns that can accompany synthetic polymer-based nanoparticles. Their natural tendency to interact with the reticuloendothelial system, and in particular with Kupffer cells lining the hepatic sinusoids, supports passive accumulation of the encapsulated payload in hepatic tissue, making them inherently suited to liver-targeted delivery even without active targeting modifications [9].

**Figure 1:**
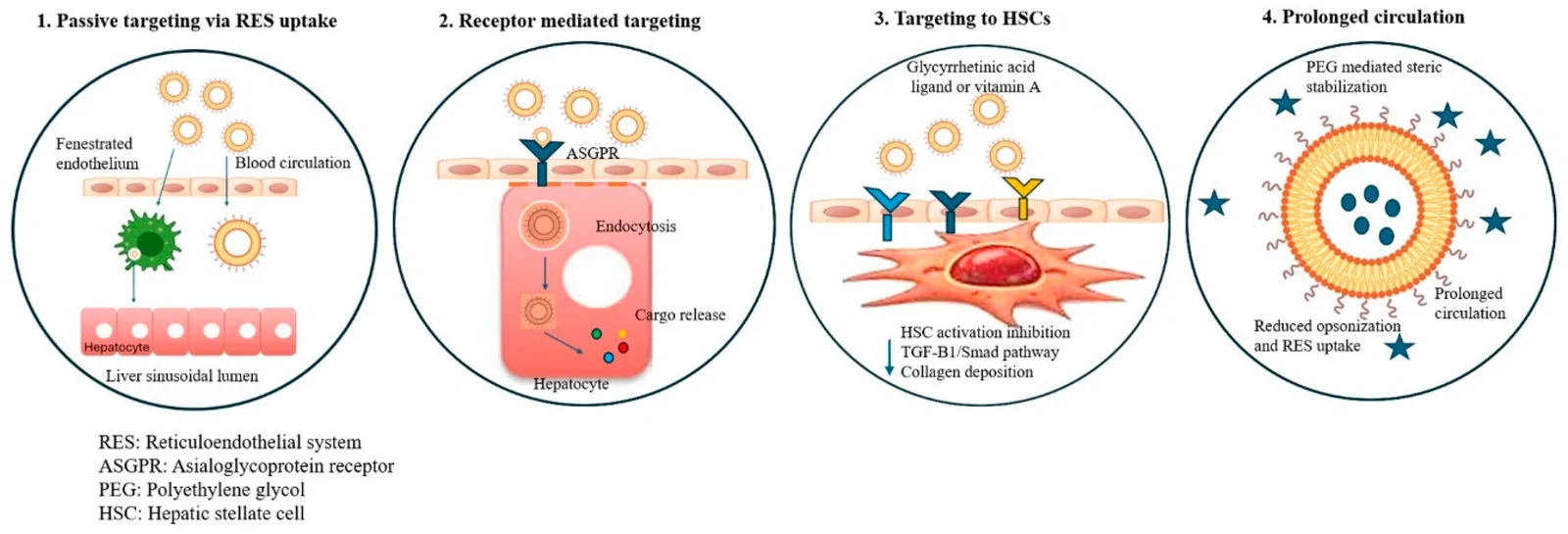
Liver-targeting mechanisms of liposomal phytochemical delivery systems. Schematic illustration of passive hepatic accumulation through reticuloendothelial system (RES) uptake, receptor-mediated hepatocyte targeting via the asialoglycoprotein receptor (ASGPR), hepatic stellate cell (HSC) targeting using glycyrrhetinic acid (GA) or vitamin A ligands, and prolonged systemic circulation achieved through PEGylation.

Conventional liposomes rely principally on passive mechanisms of hepatic accumulation, including size-dependent sequestration within hepatic sinusoids and non-specific uptake by Kupffer cells and liver sinusoidal endothelial cells, while surface-modified liposomes take a more directed approach by incorporating targeting ligands that promote selective cellular internalisation at specific hepatic cell populations. Among the ligands that have been most widely investigated for this purpose, galactose and lactobionic acid have been reported to exploit the asialoglycoprotein receptor expressed at high density on hepatocyte surfaces, glycyrrhetinic acid engages binding sites present on both hepatocytes and activated hepatic stellate cells, and vitamin A-conjugated liposomes are recognised by the retinol-binding protein uptake pathway active in stellate cells during fibrogenic activation. Furthermore, polyethylene glycol surface grafting increases the half-life of systemic circulation by decreasing the clearance by the mononuclear phagocyte system and opsonisation, which means that more of the injected liposomes reach the target cells in the liver. These surface engineering approaches significantly expand the therapeutic potential of liposomal botanical formulations, allowing for cell-type-specific delivery to hepatocytes, Kupffer cells, and activated stellate cells, depending on the modification used [10,11]. The diverse mechanisms by which liposomes achieve selective hepatic delivery, including passive accumulation, receptor-mediated targeting, and prolonged systemic circulation, are illustrated in Figure 1.

All these observations suggest that liposomal encapsulation is a promising approach to enhance the therapeutic efficacy of hepatoprotective agents of plant origin. But there are several important practical and translational issues that need to be resolved before this potential can be consistently achieved in clinical practice [9,10]. Due to the variability of botanical starting materials, which can vary in plant species, growing conditions, post-harvest processing and extraction methodology, it is a constant challenge to achieve a consistent quality of formulation from batch to batch, especially when working with herbal liposomal preparations at an industrial scale without standardised manufacturing approaches. For many of the formulations reported in the literature, information on the long-term physical and chemical stability of these systems, and the toxicological profile of the carrier components themselves remains limited. In addition, the vast majority of the evidence available comes from in vitro cell-based assays and single dose rodent pharmacokinetic studies, and direct extrapolation to human hepatic physiology is complicated by well recognised inter-species differences in hepatic blood flow, expression of metabolic enzymes and immune responsiveness. Solutions to these concerns will need to be addressed through better manufacturing standardisation, better regulatory guidance for herbal liposomal drug products, and better-designed clinical studies with appropriate biomarker endpoints [12,13].

Previous review articles can be broadly categorized into three groups. The first includes general reviews on liposome-based herbal delivery systems, which discuss a wide range of phytochemicals without focusing on specific hepatic disorders. The second comprises reviews dedicated to individual bioactive compounds, where liposomes are evaluated alongside multiple nanocarrier platforms. The third consists of nanomedicine reviews on liver diseases that consider liposomes as one of several available delivery strategies. In contrast, the present review adopts a focused approach by limiting its primary scope to true phospholipid bilayer liposomes while clearly distinguishing evidence derived from phytosome-based formulations and other non-vesicular lipid carriers. The review critically examines the relationship between phytochemical properties and formulation design, with emphasis on phytochemical classes and their biopharmaceutical limitations, liposome composition and manufacturing strategies, physicochemical characterization and stability, hepatic and cell-specific targeting, pharmacokinetic and therapeutic performance, and clinical and regulatory translation. Particular attention is given to disease-specific targeting requirements, formulation–performance relationships, manufacturing scalability, and evidence gaps that currently constrain clinical development. The available evidence is further interpreted according to its level, distinguishing in vitro, preclinical, and clinical findings. By integrating these themes within a single framework, this review aims to provide a critical and evidence-based assessment of the opportunities and limitations of liposomal delivery for hepatoprotective phytochemicals and to identify priorities for their successful clinical translation.

## 2. Search Methodology

This article is a structured narrative review. It does not claim the status of a systematic review, and no attempt has been made to pool outcomes quantitatively or to apply formal risk-of-bias scoring: the studies surveyed differ so widely in species, disease model, dose, route, endpoint and reporting completeness that a quantitative synthesis would be neither meaningful nor honest. What follows therefore describes the literature search that informed the review, the criteria by which studies were judged to fall within its scope, and the framework used to appraise the strength of the evidence they provide, so that the basis of the selection is transparent to the reader.

The literature was retrieved from PubMed/MEDLINE, Scopus and Web of Science (Core Collection), covering publications from January 2000 to March 2025, with Google Scholar used additionally to identify early-access articles and to trace forward citations. Searches were last updated in March 2025. Search strategies combined three concept blocks using Boolean operators: a vesicle block (“liposom*” OR “nanoliposom*” OR “proliposom*” OR “phospholipid vesicle”); a phytochemical block comprising individual compound names together with the generic terms “phytochemical”, “plant extract”, “herbal” and “phytoconstituent”; and a hepatic block (“liver” OR “hepat*” OR “NAFLD” OR “MASLD” OR “steatohepatitis” OR “fibrosis” OR “cirrhosis” OR “hepatocellular carcinoma”). Compound-specific searches were run separately for each phytochemical discussed, and the reference lists of relevant articles were hand-searched for further sources.

Studies were included where they reported original data on a true phospholipid bilayer vesicle, whether conventional, PEGylated, ligand-modified, stimuli-responsive or a reconstitutable liposome, encapsulating a defined phytochemical or standardised extract, with at minimum particle size and encapsulation efficiency reported alongside a hepatic in vitro, in vivo or clinical evaluation. Non-vesicular carriers, principally niosomes, solid lipid nanoparticles, nanostructured lipid carriers and phytosomes, were excluded from the primary evidence base for this reason and are cited only where explicitly identified as platform comparators or as supportive but non-equivalent clinical evidence; studies in non-hepatic disease models are likewise treated separately rather than as hepatic efficacy data. Because the evidentiary strength of the included studies varies considerably, each efficacy claim in this review is further qualified by whether it derives from in vitro work, a single or replicated in vivo hepatic model, or human data, and findings resting on a single unreplicated study are identified as such rather than generalised.

## 3. Plant-Derived Compounds in Hepatoprotection

**Figure 2:**
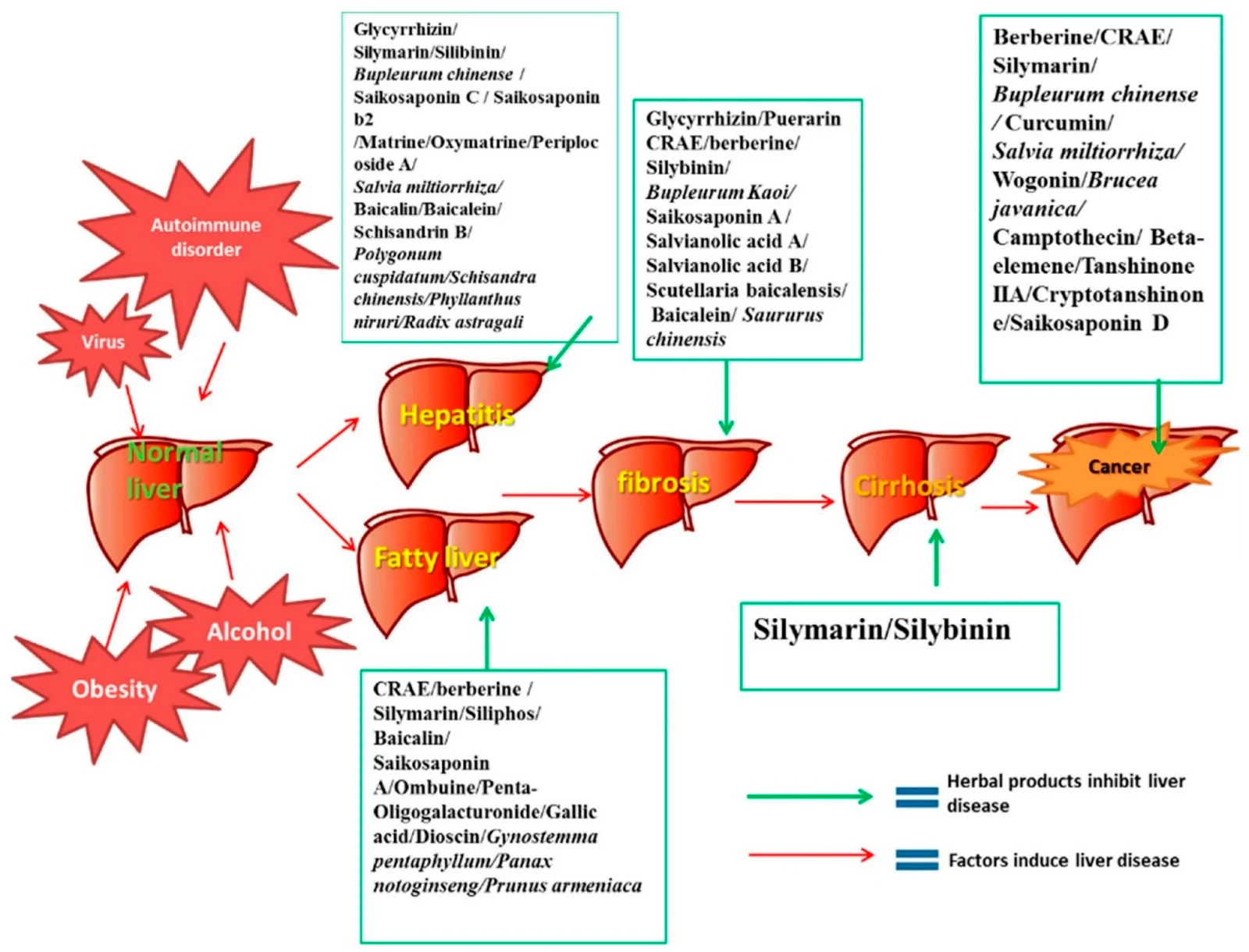
Various plants and their derived compounds for the management of chronic liver diseases adapted with permission of Hong, M et al., 2015 [18], licensee MDPI, Basel, Switzerland. This article is an open-access article distributed under the terms and conditions of the Creative Commons by Attribution (CC-BY) license 4.0.

Plant-derived hepatoprotective agents have a documented therapeutic history spanning millennia across Ayurvedic, Traditional Chinese, Unani, and indigenous botanical systems. Contemporary phytopharmacological investigation has validated many of these traditional uses at the molecular level, revealing that the hepatoprotective activity of medicinal plants is attributable to structurally diverse classes of secondary metabolites, each acting through distinct but often complementary mechanisms [14]. Based on accepted phytochemical taxonomy, these bioactive constituents can be organised into four principal categories: (i) *phenylpropanoids*, which include flavonoids (flavonols, flavanones, catechins, isoflavones) and non-flavonoid subclasses (stilbenes, lignans, phenolic acids); (ii) *diarylheptanoids*, notably curcuminoids; (iii) *terpenoids*, including monoterpenes, sesquiterpenes, diterpenes, and triterpenoids; and (iv) *alkaloids*. The plant families most frequently represented in hepatoprotective research include Asteraceae, Lamiaceae, Fabaceae, Apiaceae, and Zingiberaceae, reflecting the phytochemical richness of these lineages [15,16,17]. Table 1 and Figure 2 summarise the principal hepatoprotective plants, their key phytoconstituents, and primary mechanisms of action discussed in this review.

*Silybum marianum* (milk thistle, family Asteraceae) is one of the most extensively studied and clinically utilised hepatoprotective plants. Its pharmacologically active complex, silymarin, is a mixture of flavonolignans comprising silybin (silibinin), isosilybin, silychristin, and silydianin, with silybin being the predominant and most bioactive constituent [19]. The hepatoprotective mechanisms of silymarin are multi-dimensional: it acts as a potent antioxidant via direct free radical scavenging and upregulation of intracellular glutathione; suppresses NF-κB-mediated pro-inflammatory cytokine production (TNF-α, IL-6, IL-1β); inhibits hepatic stellate cell activation and collagen deposition via TGF-β1/Smad pathway modulation; and stabilises hepatocyte plasma membranes against toxin-induced permeabilisation. Clinically, silymarin has demonstrated beneficial effects in alcoholic liver disease, toxic hepatitis, and NAFLD in multiple randomised controlled trials, with improvements in serum ALT, AST, and hepatic histology reported at doses of 140–420 mg three times daily. Its well-established safety profile and commercial availability (Legalon^®^, Siliphos^®^) make it the benchmark hepatoprotective phytochemical against which novel formulations are typically benchmarked. However, its oral bioavailability is limited (approximately 20–50%) owing to poor aqueous solubility and extensive enterohepatic cycling, providing the primary rationale for liposomal delivery strategies [20].

*Curcuma longa* (turmeric, family Zingiberaceae) contains curcumin (diferuloylmethane) as its principal diarylheptanoid constituent, accounting for approximately 2–5% of dry rhizome weight. Curcumin exerts hepatoprotective effects through a pleiotropic mechanism that encompasses: Nrf2/ARE pathway activation with consequent induction of HO-1, NQO1, and phase II detoxification enzymes; NF-κB suppression with downstream inhibition of COX-2, iNOS, and pro-inflammatory cytokines; activation of PPAR-α to promote fatty acid β-oxidation and attenuate hepatic lipid accumulation; and modulation of apoptotic signalling via Bcl-2/Bax and caspase cascades in hepatocellular carcinoma models. In a well-characterised fructose-induced NAFLD rodent model, curcumin treatment markedly reduced insulin resistance (HOMA-IR 12.5 ± 5.4 in curcumin-treated animals vs. 39.1 ± 10.9 in disease controls), lowered serum triglycerides (163 ± 44 vs. 92 ± 21 mg/dL), attenuated hepatic protein carbonylation and lipid peroxidation (MDA: 0.27 ± 0.09 vs. 0.11 ± 0.01 nmol/mg), and restored antioxidant enzyme activities (SOD: 3.13 ± 0.83 vs. 4.90 ± 0.93; catalase: 1.27 ± 0.17 vs. 2.56 ± 0.42). Despite this broad mechanistic profile, curcumin’s clinical translation is severely constrained by its exceptionally low oral bioavailability (typically below 1%), reflecting poor aqueous solubility, photodegradation, and rapid first-pass phase II conjugation, making it a compelling candidate for bioavailability-enhancing liposomal formulation [21].

*Polygonum cuspidatum* (Japanese knotweed, family Polygonaceae) is among the richest natural sources of resveratrol (3,4′,5-trihydroxystilbene), a stilbenoid polyphenol whose hepatoprotective activity has been extensively characterised. Resveratrol attenuates fructose-induced hepatic lipid accumulation through suppression of Keap1 and activation of the Nrf2-mediated antioxidant defence pathway, reducing malondialdehyde, hydrogen peroxide, and increasing glutathione, superoxide dismutase, and catalase activities. In parallel, resveratrol favourably modulates lipid metabolism by upregulating PPAR-α expression while downregulating lipogenic transcription factors SREBP-1, FAS, and SCD-1, ultimately reducing hepatic triglyceride deposition. Beyond NAFLD, resveratrol demonstrates anti-inflammatory (SIRT1/NF-κB axis), antifibrotic (TGF-β1 suppression), and anti-tumoural activities in hepatocellular carcinoma models. Its clinical utility, however, is severely restricted by oral bioavailability consistently below 1%, arising from rapid phase II glucuronidation and sulfation, high-volume distribution, and short plasma half-life, necessitating formulation strategies that can protect the compound from presystemic metabolism while enhancing intestinal permeation [22].

*Glycyrrhiza uralensis (family Fabaceae)* is a source of the main triterpenoid saponins of hepatoprotective interest, glycyrrhizin (glycyrrhizic acid) and its aglycone, glycyrrhetinic acid (GA). Glycyrrhizin inhibits high-mobility group box 1 (HMGB1) and has antiviral activity against HBV and HCV infection, as well as anti-fibrogenic activity by inducing quiescence of HSCs [18].

GA is also of pharmacological interest as a hepatotropic ligand as the hepatocyte and activated HSC overexpress GA-binding receptors, and is therefore a therapeutic agent and a targeting agent for liposomal delivery systems as discussed in Section 4. *Phyllanthus amarus* (family Phyllanthaceae) has been shown to possess hepatoprotective and antiviral properties in hepatitis B with early clinical data, showing clearance of HBsAg in a subset of HBV carriers, and contains the lignans phyllanthin and hypophyllanthin, as well as ellagitannins and flavonoids [23,24]. *Schisandra chinensis* (family Schisandraceae) has been shown to activate Nrf2, induce CYP enzymes, and regulate P-glycoprotein, which are all hepatoprotective mechanisms, and is used for various hepatitis subtypes [25,26].

**Table 1:** Summary of the Various Plant-Derived Compound Liposomal Formulations for Liver Therapy: Methods, Characteristics, and Key Findings.

Phyto Compound	Method	Key Components	Liposomal Characteristics	In Vivo Study/ Animal Model	Pharmacokinetic Data	Key Findings/ Observations	Reference
**Flavonoids**							
**Galangin**	Thin-film hydration, Dialysis	Cholesterol, Phosphatidylcholine, Soybean lecithin	Drug release: 71.32% in simulated stomach juice (pH 1.2), 82.45% in PBS after 72 h	Sprague–Dawley rats	Liposome: Cmax: 5.31 ± 0.45 µg/mL, Tmax: 4 h; Free Galangin: Cmax: 1.25 ± 0.13 µg/mL, Tmax: 2 h	Significant increase in release compared to free drug, improved bioavailability.	[27]
**Galangin**	Thin-film dispersion	Cholesterol, DSPE-PEG, SPC	65.7 ± 6.4% (PEG-modified), 70.9 ± 3.5% (unmodified) release in 8 h	Wistar rats	PEG-modified liposomes: T1/2 about 2.7 times longer than free Galangin, 1.7 times longer than unmodified liposomes (*p* < 0.01, *p* < 0.05 respectively)	PEG-modified liposomes showed significant suppression of HepG2 cells	[28]
**Silymarin**	Reversed-phase evaporation	Dicetylphosphate (DCP), lipid, and cholesterol	Drug loading: 90%, modal size: 266–466 nm	Albino rats	Higher hepatoprotection: 55.6% vs. 33.08% of plain silymarin	Higher hepatoprotection than plain silymarin	[29]
**Quercetin**	Thin-film hydration	Phosphatidylcholine, Phosphatidylserine, Cholesterol	Improved bioactivity and bioavailability	CCl4-induced liver injury rats	Increased AUC and bioavailability in liver; specific pharmacokinetic values not reported, but significant liver protection observed	Nano liposomal quercetin protected hepatocytes and enhanced liver function	[30]
**Naringenin**	Thin-film rehydration technique	Cholesterol and Soybean lecithin	Drug loading: 8.43 ± 0.45%	Male C57BL/6 mice	AUC: 1.9-fold increase, Cmax: 6.7-fold increase in Naringenin-Nanolipo group compared to crude drug group (*p* < 0.05, *p* < 0.01)	Reduced liver lipid accumulation, enhanced absorption	[11]
**Isoliquiritigenin**	Film dispersion	Cholesterol, Sodium cholate, Soybean phospholipid	Higher cumulative release rate compared to free drug	Sprague–Dawley (SD) rats	Higher liver concentration and solubility in ISL-TPGS-PLP compared to free ISL suspension (*p* < 0.05)	Increased liver uptake and solubility, improved therapeutic effects	[31,32]
**Polyphenols**							
**Myricetin (MRC)**	Thin-film hydration	Phosphatidylcholine (soy lecithin), Cholesterol, Sodium cholate, D-α-tocopherol, Polyethylene glycol 1000 succinate	Improved oral bioavailability	Sprague–Dawley rats	Cmax, AUC (0–24), t½ values higher in MRC-PL group vs. free MRC solution (*p* < 0.05)	Hepatoprotective effect; enhanced plasma concentration with liposomal formulations, better than free MRC	[33]
**Epigallocatechin Gallate (EGCG)**	Solvent evaporation	DPPC, Cholesterol	Liposomes of 170 nm size and 0.2 zeta potential	Wistar-Bratislava rats	No specific pharmacokinetic data; improved liver protection seen in EGCG-loaded liposomes	Improved liver function, reduced histopathologic liver changes, better antioxidant regulation	[34]
**Resveratrol (RS)**	Thin-film hydration	Soy lecithin, Phosphatidylcholine, Cholesterol	145.78 ± 9.9 nm size, 78.14 ± 8.04% entrapment efficiency	Rats with liver tumours (HepG2)	AUC and Cmax increased 3.2 and 2.2 times respectively, compared to free RS (*p* < 0.05)	Higher liver tissue localization and hepatoprotective effect compared to free RS	[35]
**Psidium Extract (*P. guajava*, *P. cattleianum*)**	Thin-film hydration	Phosphatidylcholine, Cholesterol	Nano-liposomes with hepatoprotective effects	Wistar rats (paracetamol-induced liver toxicity)	No pharmacokinetic data provided, but liver enzyme markers improved, liver architecture restored	Restored liver architecture and GSH levels, better than silymarin treatment	[36]
**Gingerol (from Ginger)**	Thin-film dispersion	Cholesterol, Sodium cholate, L-α-phosphatidylcholine	Improved in vitro release profile	-	No specific pharmacokinetic data; higher plasma concentrations and internalization noted in HepG2 cells compared to free gingerol	Significant anti-tumour activity against HepG2 cells and increased internalization of gingerol	[37]
**Curcumin**	Solvent evaporation	Cholesterol, Dicetyl phosphate (DCP), Phosphatidylethanolamine (PE), PLGA	Maintained cellular ROS levels, improved liver function	Rats treated with CCl4	No specific pharmacokinetic data; significantly improved liver function and cellular antioxidant levels when compared to untreated CCl4 group (*p* < 0.0001)	Significant increase in antioxidant enzymes, reduced mitochondrial degradation, liver damage prevention	[38]
**Mangiferin (MF)**	Reverse phase evaporation	Soy phosphatidylcholine, Cholesterol	Hepatoprotective effects in liver damage models	Albino rats (ethanol-induced toxicity)	No pharmacokinetic data provided, but showed significant reduction in liver toxicity markers and improvement in antioxidant levels compared to plain mangiferin	Reduced liver toxicity markers (SGOT, SGPT, ALP), elevated antioxidant enzymes (GSH, SOD, catalase)	[39]
**Combretastatin A4 Phosphate (CA4P)**	Thin-film evaporation, co-liposome formulation	Phosphatidylcholine, Cholesterol, DSPE-PEG2,000-GA	Co-liposome formulation for enhanced cellular uptake	H22 tumour-bearing BALB/c male mice	In vivo liver imaging studies showed higher cellular uptake of Cur and CA4P; no detailed pharmacokinetic data provided	Enhanced anti-tumour effect and cellular uptake compared to free CA4P	[40]
**Terpenoid**							
Dihydroartemisinin (DHA)	Film dispersion-ultrasonication	Phosphatidylcholine (PC), PEG200, Cholesterol	DHA-loaded magnetic liposomes (MLPs)	Male mice (HNSCC tumour model)	No specific pharmacokinetic data; enhanced targeting observed with MLPs under magnetic field (*p* < 0.05)	Significant reduction in tumour volume compared to control and free DHA (*p* < 0.05)	[41]
Triptolide (TP)	Thin-film hydration	Egg yolk lecithin, DSPG, Cholesterol	Triptolide-loaded liposomes	Mice with PDXHCC tumours	No specific pharmacokinetic data; significant in vivo anticancer efficacy in PDXHCC model	Significant anti-tumour activity in both in vitro and in vivo PDX tumour models (*p* < 0.05)	[42]
Oleanolic Acid (OA)	Evaporation-sonication	Cholesterol, DSPC	Lipo-OA formulations	Male mice (alcohol-induced liver damage)	No specific pharmacokinetic data; significant reduction in liver biomarkers compared to OA alone (*p* < 0.05)	Lipo-OA exhibited superior hepatoprotective effects compared to OA alone (*p* < 0.05)	[43]
Glycyrrhetinic Acid (GA)	Film dispersion	18β-GA-DSPE, Coumarin-6, PC, Cholesterol	Coumarin-6 labelled liposomes for GA delivery	HepG2 cells	No specific pharmacokinetic data; enhanced uptake of GA via caveolae-mediated endocytosis in HepG2 cells	Passive and active diffusion of GA into cells via caveolae-dependent endocytosis	[44]
**Carbohydrate**							
Galactomannan (from *C. gilliesii*)	Liposomal formulation (phosphatidylcholine, cholesterol)	Phosphatidylcholine, Cholesterol	Galactomannan-loaded liposomes, negative zeta potential	Wistar rats (HCC model)	No specific pharmacokinetic data; in vivo reduction in liver biomarkers and gene expression linked to liposomal galactomannan use	Significant decrease in liver enzymes and gene expression (PI3K/Akt, PTEN, STAT5A) with increased antioxidant levels (GSH, GST)	[45]
**Alkaloid**							
Capsaicin (CAPS) & Curcumin (CUR)	Multifunctional liposomes (CAPS-CUR/GA&Gal-Lip)	Glycyrrhetinic acid (GA), Galactose (Gal), Phosphatidylcholine (PC), Cholesterol	Dual-targeted liposomes with GA & Gal for liver cancer targeting	Subcutaneous and orthotopic H22 tumour-bearing mice (HepG2 and HSC models)	No specific pharmacokinetic data; enhanced dual-targeting effect and tumour-specific accumulation in vivo	CAPS-CUR/GA&Gal-Lip showed better antitumor effects, less tumour angiogenesis, and ECM deposition compared to other liposomal formulations	[46]
Curcumin (CUR) & Berberine (BBR)	Ethanol injection (CUR&BBR/GA-HA-Lip)	Phosphatidylcholine (PC), Cholesterol, DSPE-PEG-GA, DSPE-PEG-HA	CUR&BBR-loaded liposomes with GA and HA for enhanced targeting	HCC mice model (BEL-7402 cells, HSCs)	No specific pharmacokinetic data; higher accumulation in tumour tissues and concurrent uptake by HSCs and BEL-7402 cells	CUR&BBR/GA-HA-Lip showed higher pro-apoptotic and anti-proliferation effects compared to free CUR (*p* < 0.05)	[47]

*Picrorhiza kurroa* (family Plantaginaceae) is used traditionally in jaundice and hepatitis and is a source of picrosides I and II and iridoid glycoside kutkin as its main hepatoprotective constituents. Mechanistically, picrosides have been shown to inhibit lipid peroxidation, restore hepatic glutathione-S-transferase and catalase activities and limit inflammatory mediator release, and efficacy in toxic hepatitis models is similar to that of silymarin [48]. The main active component of *Andrographis paniculata* (family Acanthaceae) is the diterpenoid lactone andrographolide, which inhibits NF-κB signalling, stimulates hepatocyte proliferation and has clinically relevant activity in infective hepatitis [49]. *Berberis vulgaris* and related *Berberis* species (family Berberidaceae) are major natural sources of berberine, a protoberberine alkaloid that regulates hepatic lipid metabolism through activation of AMPK and inhibition of PCSK9, and have been shown to be effective in NAFLD, with notable decreases in serum transaminases, triglycerides, and hepatic fat fraction [50].

Despite the compelling mechanistic and preclinical evidence summarised above, the clinical translation of the majority of hepatoprotective phytochemicals remains substantially limited by formulation-related constraints. Poor aqueous solubility (BCS Class II/IV classification), susceptibility to oxidative and enzymatic degradation, extensive phase I and II first-pass metabolism, and rapid plasma clearance collectively prevent the attainment of therapeutically sufficient hepatic drug concentrations following oral administration. The biopharmaceutical limitations are compound-specific but consistently severe: curcumin bioavailability is typically below 1%, resveratrol below 1%, and silymarin in the range of 20–50% with high inter-individual variability. These pharmacokinetic shortcomings, well-established in the literature, provide the primary scientific rationale for the development of lipid-based nanocarrier systems, and liposomal encapsulation in particular, as enabling delivery platforms for this class of botanical therapeutics [32].

The physicochemical characteristics of phytochemicals strongly influence their interaction with the liposomal membrane and consequently their encapsulation efficiency, release profile, and therapeutic performance. Highly lipophilic compounds such as curcumin, resveratrol, glycyrrhetinic acid, and oleanolic acid are expected to preferentially associate with the hydrophobic bilayer, whereas more hydrophilic or ionisable compounds may partition toward the aqueous core or bilayer interface. Poor aqueous solubility generally provides a strong rationale for lipid-based encapsulation, while molecular size, ionisation state, and lipid–drug interactions influence loading capacity and release kinetics. Chemical instability, including susceptibility to oxidation, hydrolysis, light, or heat, may further increase the benefit of encapsulation by providing a protective microenvironment. Importantly, these relationships are formulation-dependent; therefore, physicochemical properties should be considered together with lipid composition, the preparation method, particle characteristics, and administration route when interpreting differences in encapsulation efficiency and therapeutic performance (Table 2).

## 4. Liposome as Versatile Drug Delivery Platform

Liposomes are spherical, self-assembled colloidal vesicles composed of one or more concentric phospholipid bilayers surrounding an aqueous core. Their structure is directly analogous to biological cell membranes, reflecting the amphiphilic nature of phospholipid molecules: a hydrophilic phosphate head group faces the aqueous environment on both the inner and outer leaflets, while the hydrophobic fatty acid tails are sequestered within the interior of the bilayer. This architecture confers a fundamental therapeutic advantage: the capacity for simultaneous encapsulation of hydrophilic drugs (within the aqueous core), lipophilic drugs (intercalated within the bilayer), and amphiphilic molecules (partitioned at the water–bilayer interface), making liposomes one of the most versatile drug delivery platforms available across all classes of small molecule therapeutics, including the majority of hepatoprotective phytochemicals.

Compounds such as curcumin and resveratrol, which combine potent pharmacological activity with very low oral bioavailability and extensive presystemic metabolism, represent particularly strong candidates for formulation-based exposure enhancement, whereas silymarin and berberine have comparatively broader pharmacological and clinical backgrounds but remain limited by variable absorption and systemic exposure. Flavonoids and related polyphenols with poor aqueous solubility may also benefit from improved membrane association, protection from degradation, and controlled release following liposomal incorporation. However, the apparent differences in therapeutic efficacy across reported studies should be interpreted cautiously because formulations, doses, administration routes, disease models, and endpoints are not standardized. Thus, liposomal suitability should be judged by the convergence of pharmacological activity, magnitude of the biopharmaceutical limitation, formulation feasibility, and strength of translational evidence rather than by efficacy alone. Comparative evaluation of liposome generation for hepatic delivery are showing in Table 3. Collectively, the phytochemicals discussed in this section exhibit substantial hepatoprotective potential but differ considerably in their biopharmaceutical limitations and consequently in their rationale for liposomal delivery.

Other nanocarrier platforms, including solid lipid nanoparticles (SLNs), nanostructured lipid carriers (NLCs), polymeric nanoparticles, micelles, dendrimers, and exosome-based systems, offer distinct advantages for solubility enhancement, controlled release, or biological targeting. However, liposomes provide a particularly suitable combination of structural versatility, biocompatibility, and translational precedent for hepatoprotective phytochemicals. Their phospholipid bilayer permits simultaneous incorporation of hydrophilic and lipophilic compounds, while lipid composition and surface modification can be readily adapted for passive or receptor-mediated hepatic targeting. In contrast, SLNs and NLCs rely on a lipid matrix that may impose payload-specific loading and release constraints, polymeric and dendritic systems introduce material-dependent degradation and toxicity considerations, and exosome-based platforms present greater challenges in compositional standardization and manufacturing. Although these alternatives may outperform liposomes for specific payloads or applications, the combination of formulation flexibility, established characterization approaches, and clinical/regulatory experience provides the principal rationale for focusing this review on liposomal systems.

The therapeutic benefit of liposomal phytochemical delivery arises from a sequence of interconnected mechanisms. Encapsulation first protects labile phytochemicals from chemical and enzymatic degradation and can improve their apparent solubility and systemic exposure. Following administration, liposome size, surface composition, and PEGylation influence circulation time and hepatic biodistribution, while passive uptake by the hepatic reticuloendothelial system can promote accumulation within macrophage populations. Surface modification with receptor-specific ligands can further promote cell-selective uptake through receptor-mediated endocytosis, including ASGPR-mediated hepatocyte targeting and strategies directed toward activated hepatic stellate cells. Following cellular internalisation, membrane destabilisation, endosomal processing, or controlled release enables intracellular liberation of the phytochemical, thereby preserving or enhancing its interaction with molecular targets. Collectively, these processes can improve exposure at the relevant hepatic site and translate the intrinsic antioxidant, anti-inflammatory, antifibrotic, metabolic, or anti-tumour activity of the phytochemical into greater pharmacological efficacy.

Lipid composition is the most important factor in determining the physicochemical properties and biological activity of liposomes. Phosphatidylcholines (PCs), particularly egg-derived PC (EPC), soybean PC (SPC), hydrogenated soybean PC (HSPC), and the synthetic dipalmitoylphosphatidylcholine (DPPC) and distearoylphosphatidylcholine (DSPC), are the most widely employed bilayer-forming lipids in herbal liposome formulations owing to their established biocompatibility and regulatory acceptance. Cholesterol is added as a bilayer-stabilising agent at molar ratios of typically 30–50 mol%, filling the interstitial space between the phospholipid acyl chains, decreasing the permeability of the membrane, inhibiting the gel-to-liquid crystalline phase transition, and significantly enhancing the mechanical rigidity and drug retention during systemic circulation.

Anionic lipids such as distearoylphosphatidylglycerol (DSPG), dipalmitoylphosphatidylglycerol (DPPG) and dicetyl phosphate (DCP) provide a negative surface charge, which enhances colloidal stability through electrostatic repulsion and reduces non-specific protein adsorption. In contrast, cationic lipids (such as stearylamine, DOTAP) improve the interaction with negatively charged biological membranes, leading to a higher cellular uptake, but also to a higher cytotoxicity, which has to be carefully evaluated for hepatic applications. Liposomes can be divided based on lamellarity: unilamellar (small SUV 20–100 nm, large LUV > 100 nm), multilamellar (MLV > 500 nm) and oligolamellar (OLV). The first generation liposomes (conventional liposomes) are rapidly opsonised and cleared by the mononuclear phagocyte system (MPS), especially by Kupffer cells in the liver and macrophages in the spleen. This MPS tropism is used beneficially for the delivery of phytochemicals to the liver, but it reduces systemic circulation time. This is overcome by stealth liposomes (second generation) which are surface grafted with hydrophilic polymers, typically polyethylene glycol (PEG) at 5–10 mol% PEG-DSPE. The PEG corona provides a steric barrier that prevents the adsorption of opsonins, increases the plasma half-life from minutes to hours or days, and changes the hepatic distribution from predominantly Kupffer cell uptake to hepatocyte and sinusoidal endothelial cell uptake—an important difference for formulations targeting hepatocellular injury instead of phagocyte mediated pathology. Third generation liposomes are actively targeted liposomes that contain receptor-specific ligands on the PEG terminus or directly on the lipid surface, allowing cell-selective endocytosis at hepatocytes (via ASGPR: galactose, lactobionic acid, arabinogalactan) and/or at activated hepatic stellate cells (via mannose-6-phosphate receptor or retinol-binding protein: vitamin A conjugates) and/or at both cell types (glycyrrhetinic acid: GA). Liposomes offer a number of clinically significant benefits over other advanced drug delivery systems for hepatoprotective phytochemicals. Liposomes are made of natural or endogenous lipids and are completely biodegradable, as there are no synthetic polymer residues. In contrast to solid lipid nanoparticles (SLNs), liposomes can carry both hydrophilic and lipophilic payloads without the restrictions of a crystalline lipid matrix that may hinder the loading efficiency of amphiphilic polyphenols in SLNs. Compared with phytosomes (phospholipid complexes), liposomes provide true physical encapsulation and controlled release, achieving higher encapsulation efficiencies, affording bilayer protection against gastrointestinal degradation, and permitting intravenous administration a route of particular relevance in critically ill patients with acute liver failure. The existence of several approved liposomal products, including Doxil^®^, Onivyde^®^, AmBisome^®^, Vyxeos^®^ establishes a regulatory and manufacturing pathway that a liposomal phytochemical product could follow, together with a body of precedent on excipient acceptability, characterisation expectations and comparability assessment. It should be emphasised, however, that these approvals concern synthetic cytotoxic and antifungal agents and provide no evidence regarding the efficacy of liposomal phytochemicals. Their relevance to the present review is procedural rather than pharmacological. The disease-specific targeting strategies and their corresponding biological rationale are summarized in Table 4.

The table highlights how the relevant cellular target and ligand strategy vary across liver disease phenotypes according to the underlying pathological process and therapeutic objective.

## 5. Manufacturing Techniques for Liposomes

The selection of an appropriate manufacturing method is a critical determinant of liposome quality, encapsulation efficiency, and scalability. Each method imposes different mechanical, thermal, and chemical stresses on the lipid bilayer and the phytochemical payload, and the physicochemical properties of the drug (logP, pKa, molecular weight, thermal stability) must guide method selection. The principal techniques employed in the preparation of herbal liposomal formulations are discussed below in terms of their mechanism, advantages, limitations, and suitability for hepatoprotective phytochemicals.

The choice of liposome preparation method should be guided by the physicochemical characteristics of the phytochemical rather than by the manufacturing method alone. Lipophilic and relatively stable compounds are generally compatible with thin-film hydration or solvent-injection approaches, whereas highly hydrophilic compounds may require strategies that increase aqueous entrapment, such as reverse-phase evaporation or optimized active-loading approaches where chemically feasible. Ionisation behaviour can additionally determine whether gradient-driven loading is practical, while thermal and oxidative sensitivity may favour methods that minimize heating, prolonged organic-solvent exposure, or intensive mechanical processing. For formulations intended for clinical translation, these considerations must be balanced against particle-size control, batch reproducibility, solvent removal, process scalability, and cost. Thus, method selection represents a formulation-specific optimization rather than a universally superior manufacturing strategy (Table 5).

The thin-film hydration method (Bangham method) is the most commonly used laboratory scale method for preparation of herbal liposomes. Phospholipids, cholesterol and any lipophilic drug or targeting ligand are dissolved in an organic solvent (usually chloroform, methanol or a mixture of both) and then rotary evaporated under reduced pressure to form a uniform dry lipid film on the wall of the flask. The film is then hydrated with an aqueous buffer that contains the hydrophilic payload at or above the lipid transition temperature (Tc), which usually results in the formation of multilamellar vesicles (MLVs). The desired unilamellar size range is obtained by sequential membrane extrusion through polycarbonate filters (100–200 nm pore size), probe or bath sonication or high-pressure homogenisation. Thin-film hydration is especially suitable for lipophilic phytochemicals (curcumin, triptolide, galangin, isoliquiritigenin) as they naturally partition into the bilayer, with encapsulation efficiencies of 60–95% routinely reported. It has the main drawback of low scalability: solvent removal by rotary evaporation is difficult to implement at industrial scale and is time-consuming.

The ethanol injection method involves the dropwise addition of a lipid–ethanol solution into an excess aqueous phase under continuous stirring, causing spontaneous vesicle formation as ethanol rapidly disperses and the lipid concentration exceeds the solubility threshold. The technique is rapid, avoids organic solvent removal steps, and is amenable to continuous flow processing, making it attractive for scale-up. It is particularly suited to moderately lipophilic phytochemicals (silymarin, silibinin, berberine) and has been used to produce curcumin-berberine co-loaded liposomes in the published literature. Residual ethanol must be removed by dialysis or diafiltration. The reverse-phase evaporation method (REV) produces an inverted water-in-oil emulsion of the aqueous payload in a phospholipid-organic solvent mixture, which upon slow organic solvent removal collapses to form large unilamellar vesicles with exceptionally high aqueous-phase encapsulation efficiencies (up to 65–80% for hydrophilic compounds). REV is preferred when the objective is maximal loading of water-soluble phytochemicals or plant extracts (mangiferin, galactomannan, quercetin in aqueous solution). Its principal disadvantage is the requirement for prolonged contact between the encapsulated compound and organic solvents during emulsification, which may accelerate degradation of oxidation-sensitive polyphenols.

The proliposome approach generates a dry, free-flowing granular or powder intermediate produced by adsorbing the phospholipid-drug mixture onto a water-soluble carrier (mannitol, sorbitol, lactose) that spontaneously reconstitutes into liposomes upon hydration with aqueous media. Proliposomes overcome the physical instability associated with aqueous liposome dispersions (aggregation, drug leakage, hydrolytic degradation of phospholipids during storage) and are directly amenable to conventional tablet or capsule manufacturing. Silymarin proliposomes prepared on a mannitol carrier have demonstrated markedly improved pharmacokinetic profiles relative to bulk silymarin suspension in beagle dogs, with well-defined plasma concentration-time profiles and significantly enhanced bioavailability. For oral herbal hepatoprotective formulations intended for chronic administration, the proliposome format offers compelling practical advantages in terms of patient acceptability, shelf-life, and manufacturing flexibility. Following size reduction, critical quality attributes (CQAs) including particle size, polydispersity index (PDI, target ≤ 0.2), zeta potential (target ≤ −30 mV or ≥+30 mV for adequate colloidal stability), encapsulation efficiency (EE%), and drug loading must be rigorously characterized by dynamic light scattering (DLS), nanoparticle tracking analysis (NTA), and HPLC-based assay methods.

Advanced analytical techniques can provide complementary information beyond conventional particle-size, PDI, zeta-potential, encapsulation efficiency, and release measurements. In particular, nuclear magnetic resonance (NMR) spectroscopy offers a non-destructive approach for elucidating lipid composition, membrane organization, and lipid–drug interactions. Solution-state ^31^P-NMR can quantitatively characterize individual phospholipid components in intact liposomes and assess changes associated with PEGylation and membrane composition, whereas ^1^H-NMR can provide information on drug–lipid interactions and the chemical environment of encapsulated molecules. NMR-based analysis can therefore help determine whether a phytochemical is preferentially associated with the lipid bilayer or aqueous compartment and provide mechanistic insight into its interaction with membrane constituents. These approaches, together with complementary imaging and spectroscopic techniques, may improve understanding of liposome structure–function relationships and support more comprehensive formulation characterization and quality assessment [51,52].

An emerging and industrially significant advance in liposome manufacturing is microfluidic-based continuous flow production. Microfluidic platforms, including staggered herringbone mixers (SHMs) and T-junction devices, achieve controlled, millisecond-timescale mixing of lipid–ethanol and aqueous streams, generating liposomes of narrow size distribution (PDI < 0.1) with high batch-to-batch reproducibility that is difficult to achieve by conventional laboratory methods. This technology was central to the industrial-scale manufacture of the lipid nanoparticle (LNP) components of the Pfizer-BioNTech and Moderna COVID-19 mRNA vaccines and is now being adapted for phospholipid vesicle production [32]. For herbal liposomes, where phytochemical source variability already introduces formulation-level inconsistency, microfluidic manufacturing offers the prospect of superior physical standardization, continuous process analytical technology (PAT) integration, and GMP-compatible scale-out via parallelization of microfluidic channels. Long-term physical and chemical stability of herbal liposomes is further enhanced by lyophilization (freeze-drying) with appropriate cryoprotectants (sucrose, trehalose at 5–10% *w*/*v*), which converts the aqueous dispersion to a dry powder that reconstitutes to the original particle size distribution upon rehydration, extending shelf-life from months to years [53,54].

The physicochemical characteristics of liposomes collectively determine their in vivo behavior and therapeutic performance. Particle size influences circulation, tissue distribution, hepatic uptake, and cellular internalization, whereas a narrow PDI is important for formulation uniformity and reproducible biodistribution. Zeta potential affects colloidal stability, protein adsorption, cellular interactions, and clearance by the mononuclear phagocyte system. Encapsulation efficiency determines the fraction of phytochemical effectively incorporated into the vesicles and therefore influences the achievable dose and exposure profile. Membrane fluidity, governed by lipid composition and phase-transition characteristics, affects membrane integrity, drug retention, cellular interaction, and release behavior. Release kinetics further determine the duration and location of phytochemical exposure, with excessively rapid release potentially reducing the advantages of encapsulation and overly slow release limiting intracellular availability. Accordingly, these attributes should be optimized collectively rather than considered as independent quality parameters, because their combined effects ultimately influence biodistribution, pharmacokinetics, cellular uptake, and therapeutic efficacy.

The long-term stability of liposomal formulations is influenced by both physical and chemical degradation processes. Phospholipid oxidation, particularly in unsaturated lipids, and hydrolysis can generate degradation products such as lysophospholipids and free fatty acids, potentially altering membrane properties and accelerating drug leakage. Physical instability may additionally occur through aggregation, fusion, changes in vesicle size, and loss of membrane integrity, resulting in altered drug retention and release behavior. Therefore, storage conditions should be carefully controlled with respect to temperature, pH, light, oxygen exposure, and ionic environment. For formulations with limited aqueous stability, lyophilization can improve storage stability by reducing water-mediated degradation; however, the freeze-drying process itself may induce membrane disruption unless appropriate cryoprotectants, such as sugars, are incorporated. Stability assessment should consequently monitor changes in particle size, PDI, lipid composition, drug content, encapsulation efficiency, leakage, and release profile throughout storage to ensure preservation of the critical quality attributes of the formulation.

## 6. Encapsulating Herbal Compounds in Liposomes

The following sections critically evaluate published liposomal formulation strategies for hepatoprotective phytochemicals, organized by principal phytochemical class using accepted biosynthetic taxonomy. For each compound, the discussion covers: key formulation parameters (lipid composition, preparation method, physicochemical characterization), encapsulation efficiency and particle characteristics, in vitro drug release behavior, and in vivo pharmacokinetic or pharmacodynamic outcomes where reported. Table 1 provides a comprehensive comparative summary of all formulations discussed. Throughout, quantitative outcomes are emphasized to allow cross-formulation comparison, and mechanistic interpretations are clearly distinguished from descriptive observations.

**Figure 3:**
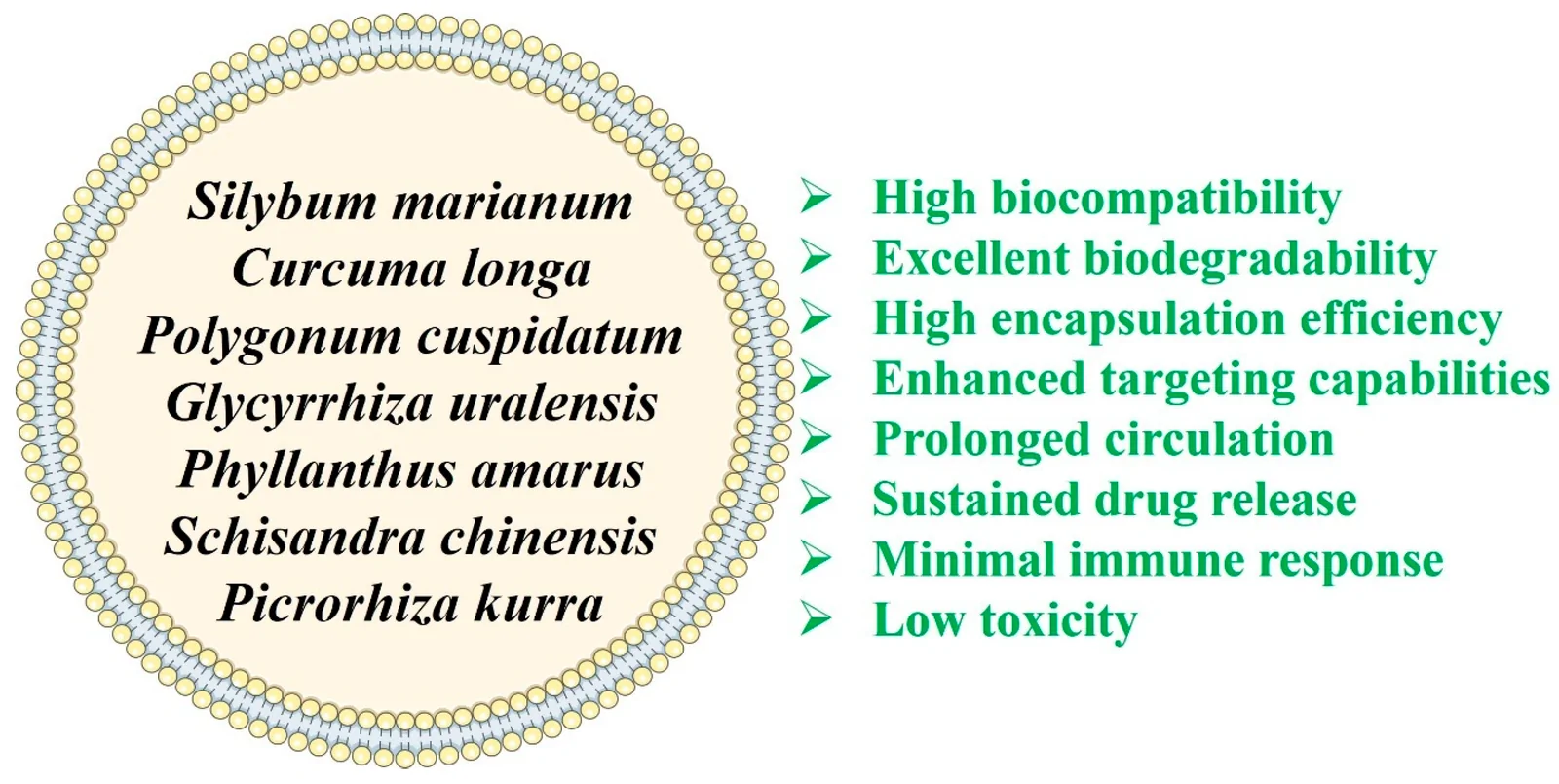
Graphical illustration of the benefits of liposomes for the delivery of plant-derived components.

Encapsulation also protects active plant molecules from enzymatic and chemical degradation and allows sustained release (Figure 3).

Table 1 provides a detailed summary of herbal liposome formulations developed for liver therapy, including preparation strategies, composition, physicochemical features, in vivo findings, pharmacokinetics, and major therapeutic outcomes.

### 6.1. Flavonoids

Flavonoids are the largest and most structurally diverse subclass of phenylpropanoid secondary metabolites, characterized by a C6-C3-C6 diphenylpropane skeleton with varying degrees of hydroxylation, methoxylation, and glycosylation. They are classified into six principal subclasses based on the oxidation state and substitution pattern of the central heterocyclic C-ring: flavonols (quercetin, kaempferol, myricetin, galangin), flavanones (naringenin, hesperidin), flavones, isoflavones, flavan-3-ols (catechins including EGCG), and anthocyanins [55]. Flavonoids exert hepatoprotective activity through multiple coordinated mechanisms: direct free radical scavenging via the catechol B-ring and C3-OH group; chelation of redox-active transition metal ions (Fe^2+^, Cu^2+^); Nrf2/ARE pathway induction with consequent upregulation of HO-1, NQO1, and glutathione-S-transferase; NF-κB pathway suppression reducing TNF-α, IL-1β, and IL-6 production; and inhibition of profibrogenic TGF-β1/Smad signalling. Despite these potent activities, the majority of flavonoids suffer from poor aqueous solubility and extensive phase II conjugation (glucuronidation, sulfation) during intestinal and hepatic first-pass metabolism, creating a compelling rationale for liposomal encapsulation [56].

Galangin (3,5,7-trihydroxyflavone), a flavonol isolated from the rhizome of *Alpinia officinarum* (Zingiberaceae), exhibits potent antioxidant, anti-inflammatory, and hepatoprotective activities, including inhibition of CYP1A1/1B1-mediated carcinogen activation and suppression of NF-κB-dependent inflammatory signalling. Its clinical translation is hampered by poor aqueous solubility and low oral bioavailability. Zhu et al. prepared galangin-loaded liver-targeting liposomes by thin-film hydration using phosphatidylcholine, soybean lecithin, and cholesterol [27]. In vitro release studies demonstrated markedly enhanced and sustained release relative to free drug suspension: liposomal galangin released 71.3% in simulated gastric fluid (pH 1.2) and 82.5% in PBS (pH 7.4) over 72 h. Pharmacokinetic evaluation in Sprague–Dawley rats confirmed a greater than fourfold enhancement in oral bioavailability (AUC), with Cmax increasing from 1.25 ± 0.13 μg/mL (free drug, Tmax 2 h) to 5.31 ± 0.45 μg/mL (liposome, Tmax 4 h), indicating prolonged absorption and improved systemic exposure [27].

PEGylated galangin liposomes were subsequently developed by Yao et al. using soybean phosphatidylcholine, cholesterol, and DSPE-PEG via thin-film dispersion, with dynamic dialysis in sodium lauryl sulfate solution for in vitro release characterisation. Both PEGylated and non-PEGylated systems exhibited biphasic controlled release—an initial rapid phase followed by sustained diffusion—contrasting with near-complete immediate release from free drug solution. Pharmacokinetic evaluation in Wistar rats demonstrated that PEG modification extended the elimination half-life approximately 2.7-fold compared to free galangin and 1.7-fold compared to unmodified liposomes (*p* < 0.01 and *p* < 0.05 respectively), consistent with reduced MPS clearance conferred by the PEG corona. Critically, PEGylated galangin liposomes showed significantly enhanced cytotoxicity against HepG2 hepatocellular carcinoma cells relative to both free drug and non-PEGylated liposomes, attributed to improved intracellular accumulation via prolonged systemic exposure [28].

Silymarin (the flavonolignan complex of *Silybum marianum*) has been incorporated into the broadest range of liposomal architectures of any flavonoid, reflecting its dual status as a benchmark hepatoprotective phytochemical and a challenging formulation subject. Elmowafy et al. prepared silymarin-loaded conventional and β-sitosterol β-D-glucoside-targeted liposomes by thin-film hydration using HSPC, cholesterol, and DSPE-PEG2000, with increasing ligand concentrations [57]. Although higher ligand density modestly reduced EE%, cellular uptake studies in HepG2 cells confirmed that non-PEGylated ligand-conjugated liposomes demonstrated the greatest internalisation, attributed to ASGPR-mediated recognition of the glucoside targeting moiety. Stability assessments confirmed physicochemical integrity for at least two months under refrigerated and ambient conditions [57].

Kumar et al. conducted a comprehensive comparison of conventional, PEGylated, anionic (DCP-containing), and cationic (stearylamine-containing) silymarin liposomes prepared by thin-film hydration with DPPC, cholesterol, and mPEG2000-DSPE. Compared to free silymarin, the conventional liposomal formulation demonstrated markedly enhanced in vitro drug release at pH 1.2 and 6.8 and superior cytoprotection in alcohol-challenged hepatocytes. In vivo, both conventional and PEGylated liposomes outperformed silymarin suspension across all pharmacodynamic and pharmacokinetic endpoints: serum Cmax increased from 100.45 ± 3.45 ng/mL (free silymarin) to 604.48 ± 14.53 ng/mL (conventional liposomes), and AUC_0–∞_ rose from 154.51 ± 7.86 to 464.37 ± 9.30 h·ng/mL an approximately fourfold improvement in systemic bioavailability. Importantly, both formulations produced significantly greater reductions in ALT, AST, IL-6, and lipid peroxidation markers, with concurrent restoration of SOD and catalase activities, compared with the suspension [7].

Silibinin (the principal pharmacologically active flavonolignan of silymarin) was encapsulated in unilamellar liposomes by ethanol injection using dicetylphosphate, phospholipids, and cholesterol, yielding particles of 266–466 nm with approximately 90% drug loading at an optimal lipid:cholesterol ratio of 10:2. In vivo hepatoprotective assessment in CCl_4_-injured albino rats demonstrated a 55.6% protective effect for liposomal silibinin versus 33.1% for the free drug—a 68% relative improvement in hepatoprotective efficacy attributable to enhanced hepatic delivery [29]. A buccal liposomal delivery system for silymarin was separately developed by El-Samaligy et al. using the reversed-phase evaporation method with soybean lecithin and cholesterol, achieving optimal sustained permeation through ex vivo chicken cheek pouch tissue for up to six hours at a lecithin:cholesterol:stearylamine:Tween 20 molar ratio of 9:1:1:0.5 [58]. The buccal route circumvents hepatic first-pass metabolism entirely and represents an underexplored delivery strategy for silymarin warranting further clinical investigation.

Co-encapsulation of silibinin and glycyrrhizic acid in PEGylated nanoliposomes (DPPC, cholesterol, mPEG2000-DSPE) was investigated by Ochi et al. to exploit the complementary anti-tumoural mechanisms of the two phytochemicals. In vitro co-delivery to HCC cells demonstrated tenfold greater cytotoxicity for the co-encapsulated PEGylated nanoliposome versus unbound silibinin (*p* < 0.05), attributed to synergistic pharmacological interaction and improved cellular internalisation of the dual payload [59]. Separately, silymarin proliposomes prepared on a mannitol carrier via film-deposition demonstrated a well-defined plasma pharmacokinetic profile following oral administration to beagle dogs, with significantly enhanced and sustained systemic exposure compared to bulk silymarin suspension—confirming that the proliposome format substantially improves the oral bioavailability of this poorly water-soluble flavonolignan complex [60].

Quercetin (3,3′,4′,5,7-pentahydroxyflavone, a flavonol) and its glycoside rutin are among the most studied flavonols for hepatoprotection, operating via Nrf2/HO-1 antioxidant pathway induction, NF-κB suppression, and direct free radical scavenging [61]. Halevas et al. characterised quercetin and rutin-loaded ceramide liposomes in a liposome-in-hydrogel composite for sustained topical or local delivery [62]. Liu et al. prepared nanoliposomal quercetin using phosphatidylcholine, phosphatidylserine, and cholesterol (5:1:1 molar ratio) via thin-film hydration combined with sonication and high-pressure homogenisation, and evaluated hepatoprotective efficacy in three complementary in vivo models: CCl_4_-induced toxic hepatitis, high-fat diet-induced steatohepatitis, and ethanol-induced acute liver injury in rats [30]. In all three models, liposomal quercetin produced significantly greater reductions in serum GPT, GOT, and direct bilirubin compared to free quercetin, with concurrent improvement in hepatic histological architecture. Histopathological assessment confirmed reduced hepatocyte ballooning, inflammatory infiltration, and lipid vacuolation in liposome-treated animals, attributable to the combined effect of enhanced hepatic quercetin accumulation and sustained intracellular drug availability.

Naringenin (4′,5,7-trihydroxyflavanone), a flavanone abundant in citrus fruits and grapefruit peel, demonstrates significant hepatoprotective activity against NAFLD through suppression of lipogenic gene expression (SREBP-1, FAS, ACC) and activation of mitochondrial β-oxidation via PPAR-α [63]. Hu et al. prepared naringenin-loaded nanostructured lipid carriers (NLC) an SLN-related platform using monostearin and soybean lecithin by hot emulsion-solidification. Naringenin-NLC achieved a drug loading of 22.5 ± 1.7%, improved in vitro release rate by 3.5-fold, significantly enhanced transepithelial transport and intestinal absorption, and increased liver distribution in rats. In a methionine-choline deficient (MCD) diet-induced NAFLD rat model, NLC-naringenin reduced hepatic lipid accumulation by approximately threefold compared to untreated controls [64]. Separately, Chen et al. prepared naringenin nanoliposomes by thin-film rehydration with soybean lecithin and cholesterol, reporting AUC and Cmax improvements of 1.9-fold (*p* < 0.05) and 6.7-fold (*p* < 0.01) respectively versus free naringenin in male mice [11]. The substantial Cmax enhancement suggests markedly improved intestinal permeation attributable to liposomal membrane fusion with enterocyte apical membranes, a mechanism of particular importance for BCS Class II/IV flavanones with intrinsically poor membrane permeability.

Isoliquiritigenin (ISL), a chalcone (an open-chain flavonoid precursor) from licorice root, has demonstrated hepatoprotective and anti-tumoural activity in HCC models through apoptosis induction and VEGF pathway suppression. Liu et al. prepared ISL-loaded TPGS-modified proliposomes (ISL-TPGS-PLP) using soybean phospholipid, cholesterol, sodium cholate, and D-α-tocopheryl polyethylene glycol 1000 succinate (TPGS) by film dispersion [41]. TPGS served dual roles as a P-glycoprotein (P-gp) inhibitor and a membrane stabiliser, addressing efflux-mediated resistance that limits intracellular ISL accumulation. In vitro release confirmed higher cumulative release for ISL-TPGS-PLP compared to unmodified ISL proliposomes and free drug suspension. Pharmacokinetic evaluation in Sprague–Dawley rats demonstrated significantly higher liver ISL concentrations for ISL-TPGS-PLP versus free ISL suspension (*p* < 0.05), whereas the unmodified proliposome did not reach statistical significance, underscoring the critical contribution of TPGS modification to liver-selective drug accumulation [31].

Myricetin (MRC) (3,3′,4′,5,5′,7-hexahydroxyflavonol) is a *flavonol* with six hydroxyl groups conferring exceptional antioxidant capacity [41]. Its hepatoprotective mechanism involves inhibition of TLR4/NF-κB/MAPK inflammatory signalling and suppression of caspase-3/9 and p53-mediated apoptotic pathways in hepatocyte injury models. Thant et al. prepared myricetin proliposomes (MRC-PL and MRC-TPGS-PL) by thin-film hydration using soybean lecithin, cholesterol, sodium cholate, and TPGS, with TPGS incorporated to enhance P-gp inhibition and membrane permeability. Dialysis-based in vitro release characterisation demonstrated controlled release from both proliposomal systems. Oral pharmacokinetic evaluation in Sprague–Dawley rats confirmed significantly higher Cmax, AUC(0–24), and t½ for MRC-PL versus free myricetin solution (*p* < 0.05). In CCl_4_-induced hepatotoxicity in mice, the high-dose MRC-TPGS-PL group demonstrated superior hepatoprotection compared to the positive silymarin control (*p* < 0.05), with greater normalisation of ALT, AST, and oxidative stress markers, representing one of the few instances where a liposomal phytochemical has been directly benchmarked against a clinical reference standard [33].

Epigallocatechin gallate (EGCG), the principal catechin of green tea (*Camellia sinensis*), is a *flavan-3-ol* classified within the flavonoid subclass of catechins, characterised by a trihydroxyphenyl B-ring and a gallate ester at C-3 that confer potent radical scavenging capacity. Bulboaca et al. prepared EGCG-loaded liposomes by solvent evaporation using DPPC and cholesterol (5:1 molar ratio), yielding 170 nm vesicles with a near-neutral zeta potential of −0.2 mV. In a gentamicin-induced hepatorenal toxicity model in Wistar-Bratislava rats, liposomal EGCG significantly improved the oxidant/antioxidant balance, reducing hepatic nitric oxide synthase activity, MMP-2 expression, TNF-α, and serum AST and ALT, while also improving renal function markers (creatinine, urea) compared to the free EGCG group. The near-neutral zeta potential of this formulation is noteworthy: while it reduces electrostatic colloidal stability, it also minimises non-specific plasma protein adsorption, which may paradoxically improve hepatic targeting selectivity by reducing premature splenic clearance [34].

### 6.2. Non-Flavonoid Polyphenols, Diarylheptanoids, and Related Compounds

The term ‘polyphenol’ encompasses all plant secondary metabolites bearing multiple phenolic hydroxyl groups across one or more aromatic rings. This broad category is correctly subdivided into flavonoids (addressed in Section 6.1), non-flavonoid polyphenols including stilbenes (resveratrol), lignans (phyllanthin), phenolic acids (gallic acid), and the structurally distinct diarylheptanoid curcumin [65].

Resveratrol (RS), a *stilbenoid polyphenol*, is produced by *Vitis vinifera*, *Polygonum cuspidatum*, and related species as a phytoalexin under oxidative stress conditions. Jagwani et al. prepared resveratrol-loaded cationic liposomes by thin-film hydration with soy lecithin, phosphatidylcholine, and cholesterol, incorporating stearylamine to generate cationic surface charge (zeta potential not specified; size 145.78 ± 9.9 nm; EE% 78.14 ± 8.04%) [33]. In vitro cytotoxicity against HepG2 hepatocellular carcinoma cells was significantly enhanced relative to free RS, attributed to cationic liposome-mediated electrostatic interaction with negatively charged tumour cell membranes enhancing internalisation. In vivo pharmacokinetic evaluation in tumour-bearing rats demonstrated AUC and Cmax improvements of 3.2-fold and 2.2-fold respectively in malignant liver tissue, with a pharmacological study confirming significantly reduced hepatocyte nodule formation and lower liver marker enzyme levels compared to the disease control [35]. It should be noted that cationic liposomes, while effective at enhancing cellular uptake, carry an established risk of complement activation, erythrocyte aggregation, and cytokine induction that must be evaluated in extended repeat-dose toxicology studies before clinical advancement.

*Psidium guajava* and *P. cattleianum* (Myrtaceae) contain a complex mixture of ellagitannins, quercetin glycosides, gallic acid, and carotenoids with established hepatoprotective activity. Saber et al. prepared nanoliposomes of standardised *Psidium* dual-species extracts by thin-film hydration with phosphatidylcholine and cholesterol, with UPLC-QTOF-MS profiling confirming phytochemical identity. In paracetamol-induced hepatotoxicity in Wistar rats, liposomal *Psidium* extract (500 mg/kg) significantly reduced serum AST, ALT, ALP, MDA, and total bilirubin, restored hepatic GSH, and normalised liver histological architecture to an extent comparable to or exceeding silymarin treatment [36,66]. This formulation is notable for encapsulating a complex botanical extract (rather than a purified single compound), demonstrating that liposomal encapsulation is feasible for standardised multi-constituent plant preparations, though the pharmacokinetic behaviour of individual constituents in such formulations warrants systematic characterisation.

6-Gingerol, the principal pungent phenylpropanoid (specifically a homovanillyl alcohol derivative) of ginger (*Zingiber officinale*), exerts anti-inflammatory, antioxidant, and anti-tumoural activity. Wang et al. prepared 6-gingerol proliposomes using L-α-phosphatidylcholine, cholesterol, and sodium cholate by the thin-film dispersion method, with dialysis-based in vitro release characterisation. The proliposomal formulation demonstrated markedly higher 6-gingerol plasma concentrations in rats compared to free drug, confirming improved oral bioavailability. Proliposomes containing 6-gingerol also showed significant anti-tumoural activity against HepG2 cells via increased intracellular accumulation, with enhanced cytotoxic potency relative to free gingerol [67].

Curcumin, a *diarylheptanoid* from *Curcuma longa*, has been the subject of the most extensive liposomal formulation research of any hepatoprotective phytochemical, reflecting both its compelling mechanistic profile and its exceptionally challenging biopharmaceutical properties [21,68]. Ali et al. prepared curcumin-loaded liposomes by solvent evaporation using phosphatidylcholine, dicetyl phosphate, and cholesterol, and compared hepatoprotective efficacy with curcumin PLGA nanoparticles in a CCl_4_-hepatotoxicity rat model [38]. Liposomal curcumin significantly maintained cellular ROS within physiological limits, increased cellular antioxidant enzyme activities (SOD, catalase, GPx), prevented mitochondrial membrane potential collapse, and reduced overall hepatic structural damage (*p* < 0.0001), outperforming the PLGA nanoparticle formulation on most endpoints [38]. This cross-platform comparison is valuable as it demonstrates the mechanistic advantage of bilayer encapsulation over polymeric entrapment for an amphiphilic polyphenol with membrane-partitioning capacity.

Dogaru et al. evaluated liposomal curcumin (DPPC, PEG2000-DSPE, cholesterol prepared by film hydration) in an acetaminophen (APAP)-induced acute liver injury model in Wistar rats, directly comparing curcumin liposomes against silymarin [60]. Liposomal curcumin produced significantly greater reductions in ALT and AST than silymarin alone (*p* < 0.01), positioning it as a superior comparator to the current clinical benchmark for paracetamol hepatotoxicity [69]. The study also demonstrated significant suppression of matrix metalloproteinase (MMP-2 and MMP-9) activity and downregulation of oxidative stress markers, suggesting that liposomal curcumin attenuates both the inflammatory and fibro-remodelling responses to APAP-induced injurya mechanistic breadth not observed with silymarin at equivalent doses. A further noteworthy formulation is the co-liposome incorporating curcumin and combretastatin A4 phosphate (CA4P) conjugated with glycyrrhetinic acid (GA)-DSPE-PEG (Cur-CA4P/GA-Lip), prepared by thin-film evaporation with L-α-phosphatidylcholine and cholesterol (3:1). CA4P, a tubulin-destabilising vascular disrupting agent, and curcumin exert complementary anti-tumoural mechanisms, and GA conjugation provides active hepatocyte and HSC targeting. The co-liposome demonstrated enhanced internalisation in BEL-7402 HCC cells, higher cytotoxic synergy, superior hepatic tissue accumulation in cell imaging studies, and significant anti-tumoural activity in H22 tumour-bearing BALB/c mice, representing an exemplary model of rationally designed combination targeted liposome therapy [40].

Mangiferin (MF), a natural plant polyphenol of C-glycosylxanthone structure from *Mangifera indica* and other sources, demonstrates hepatoprotective, antidiabetic, and antioxidant activities with potent DPPH radical scavenging capacity. Warner et al. reviewed mangiferin liposomes prepared by reverse-phase evaporation using soy phosphatidylcholine and cholesterol. In ethanol-induced hepatotoxicity in albino rats, the liposomal formulation significantly reduced SGOT, SGPT, total bilirubin, and ALP compared to free mangiferin, and restored MDA, GSH, SOD, and catalase levels toward normal values [39]. Mangiferin is a C-glucosylxanthone and therefore belongs to the xanthonoid class, structurally distinct from the flavonoids and terpenoids, although it is frequently discussed alongside polyphenols owing to its phenolic hydroxyl groups.

### 6.3. Terpenoids

Terpenoids (isoprenoids) constitute the largest and most structurally diverse class of plant secondary metabolites, biosynthesised via the mevalonate or methylerythritol phosphate (MEP) pathways from isoprene units. Hepatoprotective terpenoids span multiple structural subclasses: diterpenoids (triptolide from *Tripterygium wilfordii*), sesquiterpene lactones (dihydroartemisinin from *Artemisia annua*), pentacyclic triterpenoids (oleanolic acid, glycyrrhetinic acid), and phytosterol glucosides (β-sitosterol β-D-glucoside used as a targeting ligand) [42,70]. Their liposomal encapsulation presents unique challenges, as most terpenoids are highly lipophilic (logP > 3) with limited aqueous solubility but variable thermal stability during preparation.

Triptolide (TP), a diterpenoid triepoxide from *Tripterygium wilfordii*, demonstrates potent anti-tumoural activity in HCC at nanomolar concentrations but has an extremely narrow therapeutic window due to systemic toxicity. Yu et al. developed a photosensitiser-co-loaded liposomal system (TP/Ce6-LP) by thin-film hydration using egg yolk lecithin, DSPG, and cholesterol, combining triptolide with the photodynamic therapy sensitiser chlorin e6 (Ce6) for synergistic photoimmunotherapy. In vitro cytotoxicity against HCC cell lines and in vivo anti-tumour efficacy in patient-derived HCC xenograft (PDX) models confirmed significantly superior activity for TP/Ce6-LP over single-agent controls (*p* < 0.05), attributable to the dual mechanism of direct cytotoxicity plus light-activated reactive oxygen species generation. The use of DSPG (anionic lipid) in the bilayer conferred negative surface charge that improved colloidal stability and reduced non-specific protein adsorption, important considerations for a formulation intended for intravenous administration [42].

Dihydroartemisinin (DHA), a sesquiterpene lactone endoperoxide derived from artemisinin in *Artemisia annua*, has demonstrated iron-dependent cytotoxic activity in various cancer cell lines. Li et al. prepared DHA-loaded magnetic liposomes (DHA-MLPs) by film dispersion-ultrasonication using phosphatidylcholine, PEG200, and cholesterol, incorporating iron oxide nanoparticles to enable magnetic field-guided active targeting. In head and neck squamous cell carcinoma (HNSCC) cell lines, targeting DHA-MLPs in an applied magnetic field demonstrated significantly lower tumour volumes compared to non-targeted DHA-MLPs and free DHA in male mice (*p* < 0.05), with iron staining confirming significantly enhanced tumour accumulation under magnetic guidance [41]. It is important to note that this formulation was evaluated in HNSCC—not a hepatic disease model—and its inclusion in a review of hepatic liposomal phytochemicals requires explicit qualification. Its relevance lies in the demonstration of magnetic targeting as a physical active-targeting strategy applicable to liver tumours, where magnetic field application during image-guided interventions could be feasibly implemented.

Oleanolic acid (OA), a pentacyclic triterpenoid ubiquitous in plant cuticular waxes, demonstrates hepatoprotective, anti-inflammatory, and emerging antifibrotic activities via Nrf2 activation and TGF-β1 suppression. Wei et al. prepared liposomal OA (lipo-OA) by evaporation-sonication using DSPC and cholesterol and evaluated efficacy in a combined ambient fine particulate matter and alcohol-induced liver inflammation model in male mice. Lipo-OA produced significantly superior suppression of hepatic inflammatory markers and accelerated reparative responses compared to OA alone (*p* < 0.05), suggesting that liposomal encapsulation substantially enhanced both potency and consistency of the hepatoprotective effect [43]. The use of DSPC (Tc = 55 °C) as the principal lipid provides a gel-phase bilayer at physiological temperature, conferring high structural rigidity and prolonged drug retentionan appropriate choice for a lipophilic drug requiring extended release at hepatic sites.

*Blumea lacera* (Asteraceae) leaf extract, containing sesquiterpene lactones and flavonoids with demonstrated antioxidant and hepatoprotective properties, was formulated into liposomes by ethanol injection using egg phospholipid. In CCl_4_-induced hepatotoxicity in rats, the liposomal nanoformulation of *B. lacera* extract produced significantly lower liver biomarker levels (*p* < 0.05) and more pronounced reduction in myeloperoxidase (MPO) activity (*p* < 0.001) compared to the plain extract suspension, confirming that liposomal encapsulation of a complex botanical extract effectively enhances in vivo hepatoprotective activity relative to unformulated extract [12]. The significantly greater MPO reduction achieved by the liposomal formulation is particularly noteworthy as MPO is a key mediator of neutrophil-driven oxidative hepatitis, suggesting the liposome delivers active constituents preferentially to sites of hepatic neutrophilic infiltration.

Glycyrrhetinic acid (GA), a pentacyclic triterpenoid saponin aglycone from *Glycyrrhiza uralensis*, warrants separate discussion under targeted liposomes because of its dual pharmacological and formulation significance: it is both a hepatoprotective compound in its own right and the most widely employed active hepatic-targeting ligand in the liposome literature [44]. Sun et al. prepared coumarin-6-labelled, 18β-GA-DSPE-conjugated liposomes and characterised cellular uptake mechanisms in HepG2 cells. The study demonstrated time-dependent intracellular fluorescence accumulation at significantly higher levels than non-targeted liposomes, identifying caveolae-dependent endocytosis as the primary uptake pathway for GA-modified liposomes, in contrast to passive diffusion for unconjugated fluorophore [71]. This mechanistic clarity is significant: caveolae-mediated endocytosis leads to caveosomes that bypass the lysosomal degradation pathway, potentially preserving drug integrity after internalisation—an advantage over clathrin-mediated endocytosis for acid-labile phytochemicals.

### 6.4. Polysaccharides

Natural polysaccharides from plant, algal, microbial, and mammalian sources constitute a structurally diverse class of high-molecular-weight carbohydrate polymers with demonstrated immunomodulatory, anti-tumour, antioxidant, and hepatoprotective biological activities. Their incorporation into liposomal systems is less common than small-molecule phytochemicals, principally because their large molecular size and high hydrophilicity limit bilayer incorporation; instead, they are typically encapsulated within the aqueous core of large unilamellar or multilamellar vesicles. The following example represents the current state of evidence for liposomal delivery of hepatoprotective polysaccharides.

Galactomannan from *Caesalpinia gilliesii* seeds a high-molecular-weight β-1,4-mannan backbone with α-1,6-galactose side chains was encapsulated in liposomes prepared by varying phosphatidylcholine and cholesterol concentrations, yielding vesicles of 700 ± 100 nm with negative zeta potential and EE% ranging from 23.55 to 69.17% depending on cholesterol content. In vitro release followed a diffusion-controlled profile consistent with aqueous core encapsulation of a hydrophilic macromolecule. In an HCC rat model, liposomal galactomannan significantly reduced serum liver enzyme activities, MDA, and expression of PI3K/Akt, PTEN, and STAT5A signalling pathway genes, while increasing GSH and glutathione-S-transferase (GST) collectively indicating simultaneous antioxidant, anti-inflammatory, and antiproliferative hepatoprotective effects [45]. This formulation represents a rare example of a polysaccharide-loaded hepatoprotective liposome and demonstrates that high-molecular-weight botanical extracts can achieve therapeutically meaningful hepatic drug concentrations via liposomal delivery despite their inherent physicochemical challenges.

### 6.5. Alkaloids

Alkaloids are nitrogen-containing secondary metabolites with diverse structural classes including protoberberine alkaloids (berberine), capsaicinoids (capsaicin), indole alkaloids (vincristine), and purine alkaloids (caffeine). Among hepatoprotective alkaloids, berberine and capsaicin have been most extensively investigated in liposomal delivery systems for liver disease. Their inclusion in the same liposomal formulations with flavonoids and terpenoids (co-encapsulation strategies) also offers pharmacological rationale for synergistic combination therapy targeting complementary hepatocellular mechanisms.

Capsaicin (CAPS), the principal capsaicinoid of *Capsicum annuum*, exerts anti-inflammatory (TRPV1-mediated NF-κB suppression), anti-tumoural, and hepatic stellate cell-deactivating properties, making it a logical combination partner for curcumin in HCC therapy targeting the tumour microenvironment (TME). Qi et al. synthesised novel dual-targeted liposomes co-encapsulating curcumin and capsaicin (CAPS-CUR/GA&Gal-Lip) decorated with both glycyrrhetinic acid (GA) and galactose (Gal) as dual targeting ligands for simultaneous hepatocyte and HSC delivery. An advanced co-culture model of activated HSCs and HepG2 cells was employed to simulate the HCC tumour microenvironment in vitro. CAPS-CUR/GA&Gal-Lip demonstrated significantly superior anti-tumoural activity, reduced tumour angiogenesis, and decreased extracellular matrix (ECM) deposition compared to single-ligand (GA-only or Gal-only) liposomes and unmodified controls in subcutaneous H22 tumour-bearing mice, H22+m-HSC co-bearing mice, and orthotopic HCC models [46]. This formulation exemplifies the rational design of multi-component, multi-targeted liposomes that simultaneously address HSC-mediated stromal resistance and hepatocyte-directed tumoricidal activity a critical advance over single-compound, single-target approaches.

Berberine (BBR), a protoberberine isoquinoline alkaloid from *Berberis* and *Coptis* species, modulates hepatic lipid metabolism via AMPK activation, PCSK9 inhibition, and LDLR upregulation, with clinical evidence in NAFLD and NASH. Wu et al. prepared co-loaded curcumin-berberine liposomes (CUR&BBR/GA-HA-Lip) by ethanol injection using PC, cholesterol, DSPE-PEG-GA, and DSPE-PEG-hyaluronic acid (HA), where GA targets hepatocytes and activated HSCs via GA receptor, while HA targets CD44-overexpressing HCC cells. Biodistribution studies in an advanced HCC mouse model confirmed simultaneous tumour-tissue accumulation of both CUR and BBR and concurrent uptake by BEL-7402 cells and activated HSCs. The dual-payload GA-HA-Lip formulation demonstrated significantly greater pro-apoptotic and anti-proliferative effects in vitro and in vivo compared to free CUR alone (*p* < 0.05) and single-targeting controls, confirming additive pharmacological benefit from the CUR+BBR combination and enhanced delivery from the dual-receptor targeting strategy [47]. The concurrent targeting of two cell populations (HCC cells via CD44/HA; HSCs via GA receptor) within a single liposomal vehicle represents a mechanistically sophisticated approach to overcoming HSC-mediated stromal resistance to HCC therapy.

The available evidence indicates substantial convergence among hepatoprotective phytochemicals on Nrf2/ARE activation, NF-κB suppression, and, in fibrotic settings, TGF-β/Smad inhibition, suggesting that oxidative stress, inflammation, and fibrogenesis represent common therapeutic nodes. However, individual compounds display mechanistic features that may provide additional pharmacological differentiation. Resveratrol is more consistently associated with SIRT1-mediated metabolic regulation, berberine with AMPK-dependent lipid metabolism, glycyrrhizin with HMGB1 inhibition, and mangiferin with PPARα-associated lipid handling. Conversely, compounds such as dihydroartemisinin and triptolide exhibit mechanisms involving pro-oxidant or direct pro-apoptotic effects that differ from the antioxidant-dominant mechanisms of many polyphenols. These differences are important when considering disease-specific applications and combination strategies, although mechanistic claims should be interpreted according to the level and reproducibility of the available evidence (Table 6).

## 7. Clinical Evidence and Translational Outlook

The evidence supporting hepatoprotective phytochemicals varies substantially in strength. In vitro studies provide mechanistic and preliminary efficacy information but cannot establish hepatic pharmacokinetics or clinical benefit. Animal studies provide evidence of biodistribution, pharmacokinetic enhancement, and therapeutic effects in controlled disease models, although interspecies differences limit direct extrapolation to humans. Randomized clinical trials provide stronger evidence of therapeutic efficacy and safety, whereas systematic reviews and meta-analyses can strengthen interpretation when supported by sufficiently consistent and well-designed trials. Importantly, most evidence for true liposomal phytochemical formulations remains preclinical, while the more advanced clinical evidence largely derives from phytosome or non-equivalent phospholipid formulations and should therefore not be interpreted as direct clinical validation of liposomal systems.

The transition from compelling preclinical evidence to validated clinical utility represents the most consequential and currently least-resolved challenge in the field of liposomal phytochemical hepatoprotection. The clinical evidence base for liposomal delivery of hepatoprotective phytochemicals is, at present, limited almost exclusively to phospholipid complex (phytosome) formulations rather than true liposomal systems, reflecting the earlier commercial development timeline of the phytosome platform. Nevertheless, the clinical data available from phytosome studies provide important proof-of-concept for the bioavailability enhancement rationale and serve as the benchmark against which emerging liposomal formulations must ultimately be evaluated.

The most extensively clinically evaluated hepatoprotective phytochemical-phospholipid formulation is Realsil^®^ (silybin-phosphatidylcholine-vitamin E complex, Indena S.p.A.), a phytosome in which silybin is non-covalently complexed with phosphatidylcholine and co-formulated with vitamin E. In a multicentre, Phase III, double-blind, placebo-controlled trial (*n* = 179 patients, 138 per-protocol), Loguercio et al. evaluated Realsil^®^ administered twice daily for 12 months in patients with histologically documented NAFLD. Treatment with Realsil^®^ but not placebo produced significant improvements in liver enzyme plasma levels (normalisation of ALT, AST, and γGT over time versus baseline; *p* < 0.05 to *p* < 0.001), HOMA-IR, ultrasonographic hepatic steatosis, andin the subset of patients with paired biopsiesliver histological score. The between-group comparison was statistically significant for γGT normalisation at 12 months (*p* = 0.03), while ALT and AST showed significant within-group improvements but more variable between-group differences [72]. A subsequent systematic review and meta-analysis of four Realsil^®^ RCTs (Derakhshandeh-Rishehri et al. in 2020) confirmed a statistically significant reduction in circulating GGT (SMD = −0.37; 95% CI −0.68 to −0.06), but found non-significant pooled effects on ALT and AST across trials, underscoring the heterogeneity of the evidence base [73]. The intravenous formulation Legalon^®^ SIL (silibinin bis-hemisuccinate disodium) has been used in the emergency management of acute *Amanita phalloides* (amatoxin) poisoning, with documented case series (approximately 1500 cases) reporting overall mortality below 10% in Legalon^®^ SIL-treated patients compared with more than 20% with penicillin-based treatment alone. Its mechanism of action involves blockade of hepatic transport proteins (OATP1B1/1B3) that mediate amatoxin re-uptake, interrupting enterohepatic toxin recirculation. Although no controlled trials are available for ethical reasons, the clinical experience with Legalon^®^ SIL represents the most robustly documented use of a systemic silibinin formulation and provides important precedent for parenteral phytochemical hepatoprotection [72,73].

For curcumin, the best-characterised clinical phospholipid formulation is Meriva^®^ (curcumin-phosphatidylcholine complex, Indena S.p.A.), for which a randomised, double-blind crossover pharmacokinetic study confirmed that total curcuminoid absorption was approximately 29-fold higher than for unformulated curcumin in healthy volunteers, as published in the *Journal of Natural Products*. It is important to note that the predominant circulating forms were phase-2 metabolites (glucuronide and sulfate conjugates), and plasma concentrations remained below the levels required to inhibit most molecular anti-inflammatory targets of curcumin in vitro. Meriva^®^ has been evaluated in clinical trials for osteoarthritis, diabetic microangiopathy, and retinopathy, where improvements in clinical symptom scores and inflammatory markers have been demonstrated; however, a powered randomised controlled trial specifically evaluating Meriva^®^ in NAFLD or NASH with histological primary endpoints has not been published in the peer-reviewed literature to date. Regarding true *liposomal* curcumin, the most advanced clinical formulation is Lipocurc™ (intravenous liposomal curcumin), which has been evaluated in a Phase I dose-escalation study (ClinicalTrials.gov NCT02138955) in 32 patients with locally advanced or metastatic cancer. No dose-limiting toxicity was observed at doses from 100 to 300 mg/m^2^ per week over 8 weeks; however, haemolysis was observed in one patient at 300 mg/m^2^ over 6 h, and haemoglobin decreases were noted in three others at that dose level, identifying a safety threshold relevant for intravenous hepatic applications. No Phase II or III clinical trial of liposomal curcumin specifically targeting a hepatic indication has been registered or published as of March 2025 [33].

Resveratrol presents a more complex and contested clinical evidence picture than silymarin or curcumin formulations. Multiple meta-analyses of randomised controlled trials have produced conflicting conclusions regarding its efficacy in NAFLD, reflecting substantial heterogeneity in study design, dose (500–3000 mg/day), duration (56–180 days), and patient populations. A meta-analysis evaluated four randomized placebo-controlled trials involving 156 patients with non-alcoholic fatty liver disease (NAFLD) and found that resveratrol supplementation did not significantly improve serum alanine aminotransferase (ALT) or aspartate aminotransferase (AST) levels compared with placebo. Although modest reductions in body mass index and improvements in insulin resistance were observed in some studies, the pooled evidence did not support a significant hepatoprotective effect of resveratrol on liver enzymes, highlighting the need for larger, well-designed clinical trials to establish its therapeutic efficacy in NAFLD. The available clinical evidence therefore remains insufficient to demonstrate consistent benefits of resveratrol on hepatic biochemical outcomes [74]. A larger meta-analysis of 37 trials across multiple conditions found no significant overall change in ALT, though subgroup analysis demonstrated a significant reduction in ALT (−7.79 U/L) specifically in patients with pre-existing liver disorders [75]. Taken together, the available clinical evidence does not support a consistent, replicated beneficial effect of conventional oral resveratrol supplementation on hepatic transaminases in NAFLD, and the results should be characterised as inconclusive. This clinical equipoise strengthens, rather than weakens, the pharmacokinetic rationale for liposomal resveratrol reformulation: the oral bioavailability of unformulated resveratrol is consistently below 1%, and the negative clinical trials may reflect inadequate tissue exposure rather than true pharmacodynamic inefficacy [18,19].

A review of ClinicalTrials.gov conducted for this manuscript identified several registered trials evaluating curcumin formulations (including phytosome, nanoparticle, and solid dispersion formats) and berberine supplementation in liver disease, but no registered Phase II or III clinical trial was identified that specifically evaluates a purpose-built liposomal (bilayer vesicle) formulation of a hepatoprotective phytochemical as the primary investigational product for a hepatic indication. The Lipocurc™ Phase I trial (NCT02138955) represents the most advanced liposomal curcumin formulation in registered clinical evaluation, but was conducted in the oncology setting rather than specifically in liver disease [76]. This absence of liver disease-specific liposomal phytochemical trials reflects the current stage of the field: the translation gap between optimised laboratory formulations and Investigational New Drug (IND)-enabling regulatory packages has not been systematically bridged for any compound reviewed here. Several research directions identified in this review are anticipated to substantially advance the field in the near term. Microfluidic continuous-flow manufacturing offers the prospect of GMP-compatible, batch-consistent herbal liposome production with PDI values below 0.1, addressing the scalability limitation that has historically impeded clinical translation. Stimuli-responsive liposomes including pH-sensitive formulations triggered by the acidic hepatic tumour microenvironment, ROS-responsive lipid membranes activated at sites of hepatic oxidative stress, and phospholipase A2-triggered release in inflamed hepatic tissue—offer the prospect of on-demand drug release at the precise site of injury, further increasing the therapeutic index of potent but narrow-window compounds such as triptolide and dihydroartemisinin. Biomimetic and exosome-inspired lipid nanocarriers, including cell membrane-camouflaged liposomes derived from hepatocyte or Kupffer cell membranes, combine inherent hepatotropism with reduced immunogenicity and extended circulation, representing the next evolutionary step beyond PEGylated stealth liposomes. The gut-liver axis represents a critically underexplored dimension: liposomal phytochemicals administered orally interact with the intestinal microbiome before reaching the liver, and the metabolic biotransformation of polyphenols (equol from isoflavones, urolithins from ellagitannins, hydroxytyrosol metabolites) by gut microbiota may generate compounds with distinct or enhanced hepatoprotective activity relative to the parent phytochemical. Finally, combination liposomal strategies co-encapsulating phytochemicals with complementary mechanisms (curcumin + berberine targeting complementary metabolic and inflammatory pathways; curcumin + capsaicin co-targeting the HCC tumour microenvironment) represent a rationally designed approach to multi-target pharmacology that single-compound formulations cannot achieve [30,44,66].

**Figure 4:**
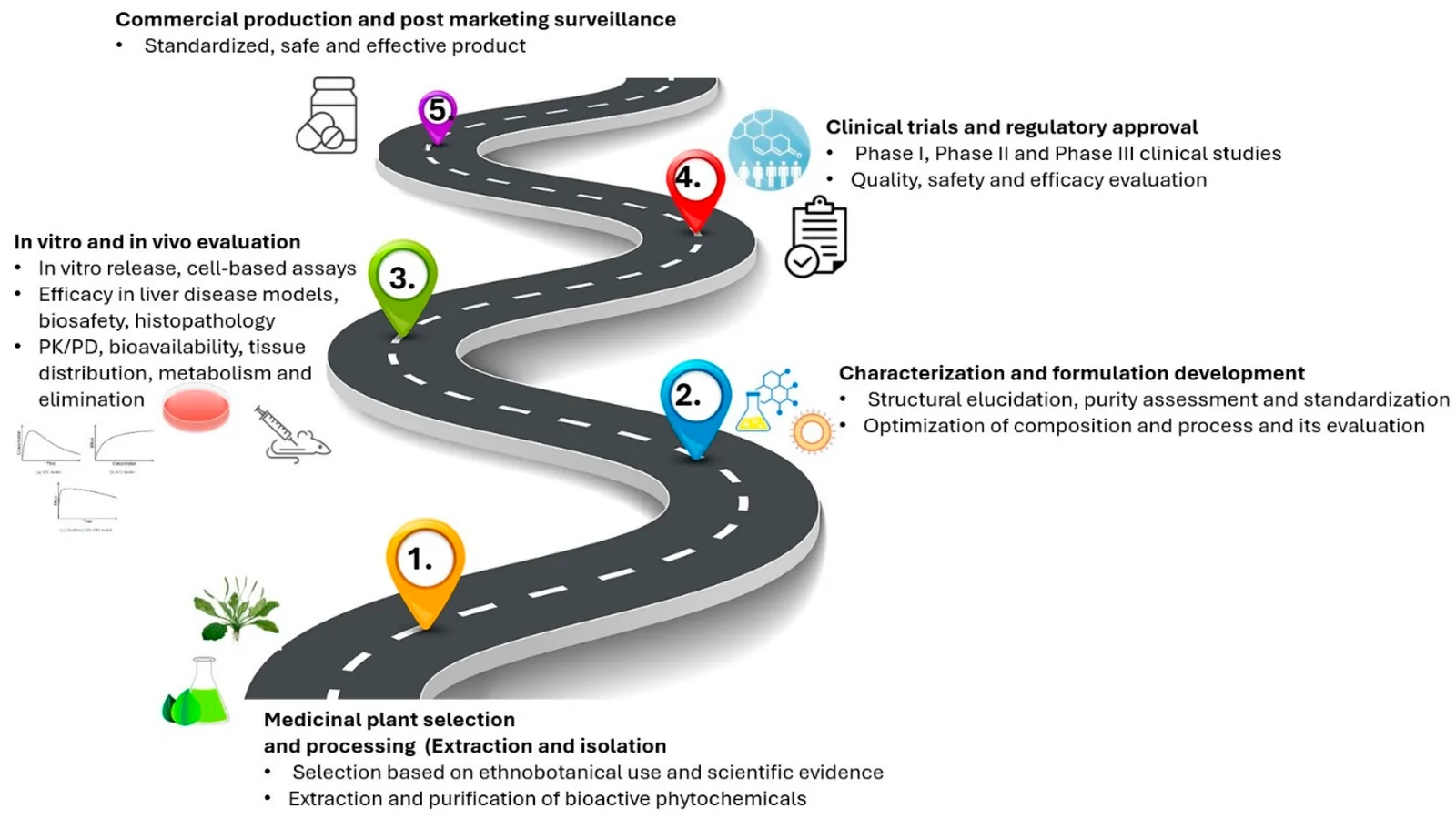
Translational roadmap for the development of liposomal phytochemical therapeutics for liver diseases. Schematic representation of the progression from medicinal plant selection and phytochemical isolation through formulation optimization, physicochemical characterization, preclinical pharmacokinetic and efficacy evaluation, clinical trials, regulatory approval, and commercial translation.

A schematic overview of the translational pipeline encompassing phytochemical discovery, liposomal formulation, preclinical evaluation, clinical development, and commercialization is presented in Figure 4.

From a regulatory perspective, comprehensive physicochemical characterization is essential for establishing the quality and performance of complex liposomal drug products. The FDA recommends assessment of liposome morphology and, where applicable, lamellarity, surface characteristics, particle size and distribution, drug encapsulation efficiency and loading, phase-transition temperature, in vitro drug release, leakage during shelf life, and changes in liposome integrity under relevant pH, ionic strength, temperature, and excipient conditions. Drug distribution within the liposomal structure should also be considered, as hydrophilic compounds are generally associated with the aqueous compartment whereas hydrophobic compounds may be incorporated within the lipid bilayer. Spectroscopic or other analytical approaches can further support evaluation of liposome structure and integrity. Collectively, these attributes are important for defining critical quality attributes and ensuring formulation consistency, stability, and predictable drug-release behaviour during development and commercialization [77].

## 8. Conclusions

In conclusion, liposomal delivery systems represent a versatile and mechanistically well-characterised platform for improving the biopharmaceutical performance of hepatoprotective phytochemicals. The convergence of improved biopharmaceutical performance, cell-selective hepatic targeting, intracellular mechanism preservation, and an established regulatory framework positions herbal liposomes as credible candidates for clinical development in chronic liver disease—provided that the field collectively addresses the manufacturing, standardisation, toxicological, and clinical evidence gaps identified in this review. Progress in these areas, guided by rigorous quality-by-design principles, biorelevant preclinical models, and well-designed proof-of-concept clinical pharmacokinetic studies, will be essential to realise the therapeutic potential that the preclinical literature compellingly, if incompletely, demonstrates.

Emerging approaches integrating artificial intelligence and machine-learning-assisted formulation optimization, digital formulation platforms, and Quality by Design (QbD) principles are expected to further accelerate the rational development of liposomal phytochemicals by enabling prediction and optimization of critical formulation attributes and process parameters. In parallel, continuous manufacturing and process analytical technologies may improve batch-to-batch consistency, scalability, and real-time quality control, thereby addressing important barriers to the industrial translation of complex herbal liposomes. The integration of these digital and manufacturing technologies with mechanistic modelling and robust regulatory characterization could facilitate a more predictable and scalable pathway from laboratory formulation to clinically relevant liposomal therapies.

## Figures and Tables

**Table 2:** Physicochemical determinants of liposomal encapsulation and delivery of representative hepatoprotective phytochemicals.

Compound	MW (g/mol)	Lipophilicity	Aqueous Solubility	Chemical Stability	Expected Predominant Localization	Formulation Implication
Curcumin	368.4	High	Very low	Light/oxidation sensitive	Bilayer/interface	High rationale for lipid encapsulation; protection and sustained release
Silibinin	482.4	Moderate	Low	Moderate	Bilayer/interface	Lipid incorporation may improve dissolution and exposure
Resveratrol	228.2	High	Low	Light/oxidation sensitive	Bilayer	Encapsulation may improve stability and systemic exposure
Quercetin	302.2	Moderate	Very low	Oxidation sensitive	Interface/bilayer	Solubility enhancement and protection from degradation
EGCG	458.4	Low	Relatively higher than many flavonoids	Oxidation/thermal sensitive	Interface/aqueous–bilayer boundary	Limited membrane partitioning may require formulation optimization
Berberine	336.4 (free base)	Ionisation-dependent	Salt-dependent	Relatively stable	Aqueous phase/interface	Ionisation and pH should guide loading strategy
Glycyrrhetinic acid	470.7	High	Very low	Relatively stable	Bilayer	Strong rationale for bilayer incorporation
Oleanolic acid	456.7	Very high	Extremely low	Relatively stable	Bilayer	Liposomal delivery can address poor aqueous solubility
Triptolide	360.4	Moderate	Low	Chemically sensitive	Bilayer/interface	Encapsulation may improve exposure but stability and toxicity require careful control
6-Gingerol	294.4	Moderate–high	Low	Thermally sensitive	Bilayer	Lipid incorporation may improve stability and controlled release
Mangiferin	422.3	Low/moderate	Moderate	Relatively stable	Aqueous/interface	Hydrophilicity may favour aqueous or interfacial localization

**Table 3:** Comparative evaluation of liposome generations for hepatic delivery.

Generation	Design Rationale	Dominant Hepatic Distribution/Target	Circulation Half-Life	Principal Advantages	Principal Limitations	Representative Example	Current Clinical Applicability
Conventional (unmodified)	Bare phospholipid bilayer, no surface modification	Kupffer cells via RES/MPS uptake	Minutes to ~1–2 h	Simple manufacture; exploits natural hepatic tropism for macrophage-driven disease,	Rapid opsonisation and clearance; unsuitable where prolonged circulation or hepatocyte access is required	Galangin liposomes (Section 6.1)	Preclinical; suited to alcohol-related and inflammatory liver disease models
PEGylated (stealth)	PEG-DSPE grafted at 5–10 mol% forms a steric corona	Redirected from Kupffer cells toward hepatocytes and sinusoidal endothelium	Hours to days	Reduced opsonin adsorption; markedly extended half-life; improved access beyond the sinusoid	Reduced cellular uptake and fusogenicity (“PEG dilemma”); anti-PEG antibody risk on repeat dosing	PEGylated galangin liposomes (Section 6.1)	Preclinical for phytochemicals; platform approved for synthetic payloads (Doxil)
Ligand-targeted (third generation)	Receptor-specific ligand on PEG terminus or lipid headgroup	Cell-selective: hepatocyte (ASGPR), stellate cell (RBP, M6P/IGF-II R), or both (GA)	Comparable to PEGylated, modestly reduced by ligand density	Cell-selective uptake; can address a specific pathological population rather than bulk liver	Requires defined and controlled ligand density; manufacturing complexity; valency-dependent efficacy	GA-modified curcumin/berberine liposomes (Section 6.5)	Preclinical only for hepatoprotective phytochemicals
Biomimetic (cell-membrane coated)	Coating with hepatocyte, erythrocyte or exosomal membrane fragments	Determined by source membrane; potential for homotypic or immune-evasive targeting	Variable; erythrocyte-coated systems report extended circulation	Native membrane proteins confer immune evasion and, in principle, homotypic targeting without synthetic ligand chemistry	Manufacturing complexity and batch-to-batch membrane variability; not yet applied to a hepatoprotective phytochemical in this review	Not represented among formulations reviewed here	Emerging; no hepatoprotective phytochemical example identified in this literature
Stimuli-responsive	Bilayer components sensitive to pH, redox, enzyme or temperature trigger payload release	Determined by the accompanying targeting strategy; release is triggered locally	Comparable to the underlying generation (conventional, PEGylated or targeted)	Site-selective release reduces off-target exposure; can be combined with any of the above generations	Adds formulation and characterisation complexity; trigger specificity must be validated in the intended disease microenvironment, not only in vitro	Not represented among formulations reviewed here	Emerging; a plausible next step for the ligand-targeted systems

**Table 4:** Disease-specific targeting strategies and biological rationale for ligand-mediated liposomal delivery in liver diseases.

Disease Phenotype	Relevant Target Cell	Ligand	Receptor/Target	Uptake Mechanism	Potential Therapeutic Application	Principal Limitation
Viral hepatitis (HBV/HCV)	Hepatocytes	Galactose/lactobionic acid	ASGPR	Receptor-mediated endocytosis	Hepatocyte-directed intracellular delivery	Hepatocyte uptake does not necessarily ensure delivery to the relevant intracellular viral compartment
Alcohol-related liver disease	Kupffer cells/hepatocytes	Mannose/conventional liposomes	CD206/RES-MPS	Receptor-mediated or passive hepatic uptake	Macrophage-associated inflammatory pathology	Cellular target may vary with inflammatory and metabolic features
MASLD/MASH	Hepatocytes/Kupffer cells	Galactose/lactobionic acid/mannose	ASGPR/CD206	Receptor-mediated endocytosis	Metabolic and inflammation-directed delivery	Relevant target may vary with disease phenotype and stage
Fibrosis/cirrhosis	Activated HSCs	Vitamin A/M6P	RBP/STRA6/M6P–IGF-II receptor	Receptor-mediated uptake	Antifibrotic delivery to activated HSCs	Fibrotic remodeling and sinusoidal capillarization may restrict access
Hepatocellular carcinoma	Tumour cells/stromal cells	Glycyrrhetinic acid/hyaluronic acid	GA-binding sites/CD44	Receptor-associated endocytosis	Tumour- and microenvironment-directed delivery	Tumour heterogeneity and altered hepatic architecture may limit reproducible targeting

**Table 5:** Comparative evaluation of liposome preparation strategies and their suitability for phytochemical delivery.

Method	Encapsulation Efficiency	Size/PDI Control	Scalability & Reproducibility	Solvent/Process Considerations	Relative Cost	Thermal/ Mechanical Stress	Best Suited to
Thin-film hydration	Moderate–high for suitable lipophilic compounds; generally lower for hydrophilic compounds	Broad size distribution unless followed by extrusion	Limited scalability; operator-dependent	Organic solvent removal required	Low at laboratory scale	Low–moderate	Lipophilic, relatively stable phytochemicals
Ethanol injection	Moderate–high for suitable lipophilic compounds	Better control than conventional hydration; further size reduction may be required	Relatively amenable to scale-up/continuous processing	Ethanol; solvent removal may be required	Low–moderate	Low	Moderately lipophilic compounds
Reverse-phase evaporation	Moderate–high; useful for hydrophilic payloads	Relatively broad without downstream size reduction	Moderate; scale-up can be challenging	Greater organic-solvent exposure	Moderate	Moderate	Hydrophilic or amphiphilic phytochemicals
Proliposome	Dependent on parent preparation method	Dependent on reconstitution	Good potential for dry-state manufacturing	Reduced solvent in final dry product	Low–moderate	Low	Compounds requiring improved storage/solid-state stability
Microfluidic mixing	High and reproducible for suitable formulations	Excellent control with narrow distributions achievable	High potential for continuous and scalable manufacture	Typically low-boiling/acceptable organic solvent systems; downstream removal required	Higher equipment cost	Low–moderate	Formulations requiring tight size control and scale-up
Remote/active loading	Potentially high for suitable ionisable compounds	Preformed vesicle characteristics largely retained	Scalable when compatible with the drug and gradient	Depends on vesicle preparation and loading system	Moderate	Low	Ionisable compounds amenable to gradient-driven loading

**Table 6:** Comparative molecular mechanisms of major hepatoprotective phytochemicals.

Compound	Nrf2/ARE	NF-κB	TGF-β/Smad	AMPK	SIRT1/PPARα	Principal Differentiating Mechanism
Silymarin/silibinin	↑	↓	↓	–	PPARα	Membrane stabilization and multidimensional hepatoprotection
Curcumin	↑	↓	↓	↑	PPARα	Broad pleiotropic antioxidant, inflammatory and metabolic modulation
Resveratrol	↑	↓	↓	↑	SIRT1	SIRT1-associated metabolic and inflammatory regulation
Quercetin/EGCG	↑	↓	↓	–	–	Direct antioxidant and metal-chelating activity
Berberine	↑	↓	–	↑↑	–	AMPK activation and lipid-metabolic regulation
Glycyrrhizin/GA	↑	↓	↓	–	–	HMGB1 inhibition and anti-inflammatory activity
Mangiferin	↑	↓	–	–	PPARα	Potentially relevant to hepatic lipid handling
Triptolide	–	↓	↓	–	–	Direct pro-apoptotic/antifibrotic activity; narrow therapeutic window
Dihydroartemisinin	–	–	–	–	–	Iron-dependent pro-oxidant/apoptotic mechanism

## Data Availability

No new data were created or analyzed in this study. Data sharing is not applicable to this article.
